# Cultivation of *Limnospira platensis* (Spirulina) in Full Seawater with Medium Recycling: A Promising Source of Protein and Phycocyanin for Arid Coastal Regions

**DOI:** 10.3390/md24040141

**Published:** 2026-04-16

**Authors:** Monserrat Alemán, Marianna Venuleo, Juan Luis Gómez-Pinchetti, Eduardo Portillo, Flavio Guidi

**Affiliations:** 1 Instituto Tecnológico de Canarias (ITC), Biotechnology Department, Playa de Pozo Izquierdo s/n, 35119 Santa Lucía de Tirajana, Las Palmas, Spain; mvenuleo@itccanarias.org (M.V.); eportillo@itccanarias.org (E.P.); fguidi@itccanarias.org (F.G.); 2 Banco Español de Algas (BEA), Instituto de Oceanografía y Cambio Global (IOCAG), Universidad de Las Palmas de Gran Canaria, Muelle de Taliarte s/n, 35214 Telde, Las Palmas, Spain; juan.gomez@ulpgc.es

**Keywords:** *Limnospira*, seawater medium recycling, long-term cultivation, freshwater scarcity, energy and nutrient efficiency, alternative proteins, phycocyanin, food-grade biomass, environmental sustainability

## Abstract

Protein and phycocyanin production is challenged by freshwater scarcity in arid coastal regions. This study assessed and optimized the cultivation of *Limnospira platensis* BEA 1257B in full seawater. Eight cultivation phases were conducted in 10,000 L raceways under a greenhouse to evaluate the effects of seawater content, nutrient availability, shading, CO_2_ supply, and medium recycling on biomass productivity and biochemical composition. Freshwater, energy, and fertilizer savings, together with effluent characteristics of the optimized full-seawater recirculation strategy (SWR), were evaluated against a conventional freshwater cultivation process. Lower productivity was associated with high salinity and irradiance. Under long-term optimized conditions (615 days), the strain achieved stable productivities of 4.1 ± 1.4 gDW m^−2^ day^−1^ (14.8 ± 5.0 tDW ha^−1^ year^−1^). Increasing salinity promoted carbohydrate accumulation in the biomass (26.0% AFWD), while protein (64.4%) and C-phycocyanin (9.9%) moderately decreased. Nevertheless, protein quality, phycocyanin, and essential fatty acids remained high. Spray-dried biomass exhibited nutritionally relevant contents of K, Mg, Ca, Fe, and Mn, and complied with international food safety standards. SWR reduced energy demand by 10.5% and freshwater consumption by 12% on a surface basis, although these advantages were partially offset when expressed per unit of product, while clearly supporting environmentally sustainable and regulatory-compliant *Limnospira* production.

## 1. Introduction

Global agriculture faces challenges due to increasing freshwater scarcity, land degradation, and the need to sustain a growing population, particularly in arid and semi-arid regions, which account for nearly 40% of Earth’s landmass [1,2]. Ensuring sustainable food sources of vegetable origin in the coming decades is a challenge that requires novel approaches to strengthen food security while reducing reliance on freshwater resources [3].

The cultivation of the extremophile cyanobacterium *Limnospira platensis* (syn. *Arthrospira platensis*) offers a viable biomass production option due to its ability to thrive in alkaline and brackish environments, which allows its cultivation on non-arable land using alternative water sources [3,4]. Moreover, *L. platensis* is known for its high protein content (typically 50–70% of dry weight in commercial products), essential amino acids, minerals, vitamins, pigments, and other antioxidants [5,6,7], making it a feasible choice for reducing malnutrition in underdeveloped regions [5], as well as for its applications in functional foods and beverages, and dietary supplements [8,9]. Among the high-value compounds synthesized by *Limnospira*, the blue pigment phycocyanin (up to 25% of dry weight [10]) is a phycobiliprotein highly valued in the food, cosmetic, and pharmaceutical industries due to its coloring, antioxidant, anti-inflammatory, and neuroprotective properties [11].

Nevertheless, approximately 1 m^3^ of desalinated water is typically required to produce just 1 kg of dry microalgal biomass, which has intensified interest in reducing freshwater dependency in large-scale cultivation systems [12]. The use of seawater as an alternative water and nutrient source for *Limnospira* cultivation has been previously investigated as a sustainable method for biomass production in freshwater-limited environments [13,14,15], demonstrating how partial or complete substitution of traditional freshwater-based with seawater-based culture media can reduce production costs by up to 50% [16]. However, several studies report difficulties in sustaining consistent biomass productivities and protein and phycocyanin contents under saline conditions [12,15,16,17]. Additionally, sodium accumulation in the biomass remains a key concern regarding the use of marine microalgae for food applications [3,12].

While supplementation of limited amounts of seawater as a source of minerals and trace elements to freshwater culture media has already been implemented in the industrial cultivation of *Limnospira* by Cyanotech Corporation (Kailua-Kona, HI, US), most studies on *L. platensis* cultivation in high salinity or full-seawater-based media have been conducted under controlled laboratory conditions [18,19,20,21], with only a few outdoor trials [12,22]. One small-scale outdoor experiment reported mean annual productivities of 7.35 gDW m^−2^ day^−1^ for *L. maxima* cultivated in seawater enriched with urea over a few days [23], while *L. maxima* cultivated at a pilot scale in Saudi Arabia reached productivities of up to 60 gDW m^−2^ day^−1^ in open raceway ponds (RWs) under extreme irradiance and temperature conditions [24]. A recent 1-month pilot-scale study in southern Spain achieved productivities of 16 ± 1.9 gDW m^−2^ day^−1^ for *L. platensis* cultivated in seawater in greenhouse-covered RWs [12]. Despite these advances, no previous studies have reported the year-long pilot-scale cultivation of *L. platensis* using 100% seawater, nor the long-term recirculation of a seawater-based medium for this cyanobacterium.

Beyond the use of seawater, reducing nutrient use has been identified as a key strategy to further decrease production costs and minimize environmental impacts [15,25,26]. In this regard, optimizing macronutrient supply to the make-up culture medium (i.e., nitrate, phosphate, and carbonate) is crucial to reduce production costs and dependence on fertilizer imports, while also ensuring compliance with effluent discharge regulations that require adherence to established physicochemical quality standards—an aspect that is rarely considered [26]. A recent study revealed N–NO_3_ and P–PO_4_ concentrations in the effluent reached 181 and 20 mg L^−1^, respectively, exceeding local discharge limits by approximately nine-fold and two-fold [27].

From an economic perspective, RWs are the most feasible systems for *Limnospira* cultivation due to their simple design, easy scalability to large volumes, and low capital investment and operational costs. In these systems, biomass productivity is mainly determined by geographical location, light intensity, pond depth, CO_2_ supply, culture mixing, and medium type [28]. Additionally, cultivation under greenhouses in subtropical arid and semi-arid areas improves both control of operational parameters and biomass quality by mitigating excessive irradiance and UV stress, buffering temperature fluctuations, and reducing exposure to wind-borne dust and external contaminants, thereby promoting the production of stable, food-grade biomass [3,29].

This study aims to assess the technical feasibility of acclimating *Limnospira platensis* to a full-seawater-based, recirculated culture medium and producing it in semi-arid subtropical coastal regions, while successively optimizing the process to achieve stable productivities and consistent protein and phycocyanin contents in the biomass, while ensuring compliance with effluent discharge limits. To the best of our knowledge, this is the first study conducted in the Canary Islands using a *Limnospira* strain cultivated in seawater, and the first worldwide to implement long-term cultivation with medium recirculation in full-seawater conditions within an integrated, sustainability-focused framework. The findings underscore the potential of *Limnospira platensis* to serve as a cornerstone of marine-based biotechnologies, enabling the development of long-term sustainable food systems in coastal regions with limited agricultural resources and freshwater scarcity, thereby contributing to the alleviation of nutritional deficiencies and improving food security.

In this context, the Canary Islands offer ideal conditions for year-round outdoor microalgae cultivation due to their stable climate and high solar irradiance [3,30,31], together with the availability of non-arable land, access to high-quality seawater, and a strategic location, where *L. platensis* is allowed for cultivation under the Regional Plan for Aquaculture (PROAC; [32]). A recent pilot-scale study performed in the southeast of Gran Canaria island demonstrated the feasibility of long-term cultivating the local strain *Limnospira platensis* BEA 1257B in RWs under greenhouse conditions using a freshwater-based medium, yielding stable biomass productivity (6 gDW m^−2^ day^−1^, corresponding to 21.9 tons ha^−1^ year^−1^) and a consistent biochemical profile (>60% protein and >7% C-phycocyanin in dry weight). The semi-continuous cultivation at low culture depth (0.10 m) with medium recycling greatly decreased freshwater use; however, the environmental implications of the culture medium used, effluent management, and their regulatory compliance were not taken into account [3].

Although the term Spirulina is widely used in commercial contexts and in part of the literature for this cyanobacterium, taxonomic revisions have reassigned the cultivated species to the genus *Limnospira* [33,34], whose use is adopted in this study.

## 2. Results

### 2.1. Environmental Parameters and Culture Conditions

The average daily solar radiation (G_0_), culture temperature, pH, salinity, CO_2_ supply rate, evaporation rate (ER), and initial and final culture concentration (Cx) at each semicontinuous cycle for each cultivation phase are reported in Table 1.

**Figure A1:**
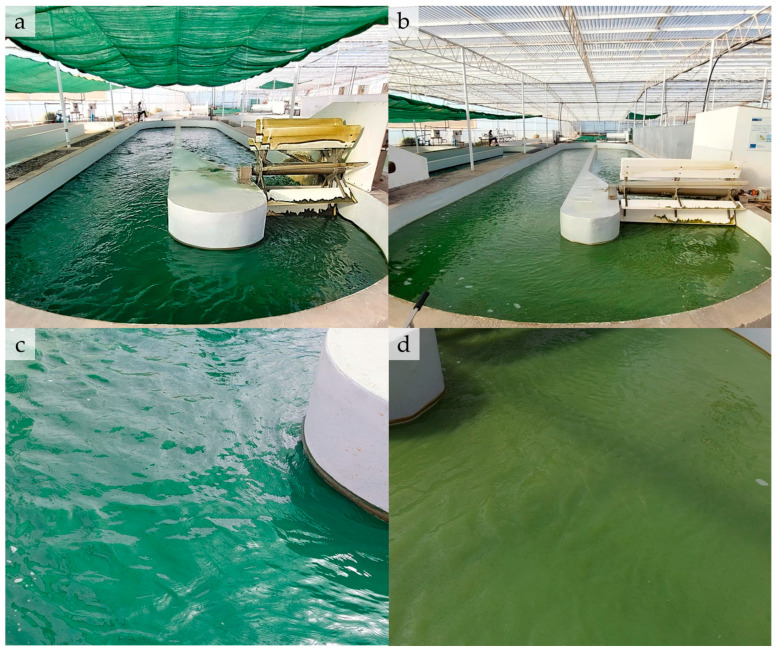
Outdoor cultures of *L. platensis* BEA 1257B in full-seawater under a greenhouse in 80 m^2^ RWs. The images show different visual appearances of the culture observed during the same cultivation period: change in color from blue-green under screen-shaded conditions (**a**) to pale-green under no screen-shaded conditions (**b**); change in culture appearance from blue-green (**c**) to milky green (**d**) both under shaded-screen conditions.

Throughout the entire cultivation period, the average full G_0_ was 247.36 ± 71.88 W m^−2^ day^−1^. However, it must be taken into account that for all the experimental phases, full G_0_ was attenuated by approximately 66% before hitting the culture (28% from the greenhouse and a further 53% from the shading screen). The only exception was the P6NS phase, where no shading screen was used and attenuation was only due to the greenhouse structure. The reason of comparing the growth performance of a screen-shaded culture with a non-shaded culture during the same period (Table 2) was that, in previous attempts to remove the shading screen, culture pigmentation shifted from its characteristic blue-green color to a pale-green one in 24–48 h (Appendix A.1, Figure A1), while after re-installing the shading screen, the culture gradually turned back to its original color in a few days. Corrected G_0_ values across all phases ranged from 6 to 250 W m^−2^ day^−1^, with a mean value of 84 ± 24 W m^−2^ day^−1^. As expected due to seasonal variation, P2 and P5 (105 ± 18 and 101 ± 20 W m^−2^ day^−1^, respectively) exhibited significantly higher irradiance values compared to P1, P6_Total_, and particularly P4, which had the lowest recorded irradiance (64 ± 17 W m^−2^ day^−1^, *p* < 0.05; Dunn’s post hoc test). As expected, P6NS exhibited the highest corrected G_0_ (161 ± 52 W m^−2^ day^−1^).

Temperature during long-term cultivation ranged between 14.9 and 34.6 °C; however, the average culture temperature (22.7 ± 2.7 °C) varied within a very narrow interval across phases (22.0–25.0 °C), with the significantly highest values observed in P2, P3, P5, and P7CO_2_^+^ and the lowest in P4 and P6_Total_ (*p* < 0.05; Dunn’s post hoc test), which were approximately 9% lower (Table 1).

The pH showed relatively little variation with an overall mean of 9.5 ± 0.5. However, a decreasing trend in pH was observed with increasing culture salinity, since higher pH values were recorded during the 10% seawater phases P1 and P2 (10.14 ± 0.3 and 10.24 ± 0.3, respectively) compared to the experimental phases with the full-seawater medium (P4 onwards: 9.39 ± 0.37, *p* < 0.05; Dunn’s post hoc test, Table 1).

Salinity was the lowest in P1, P2, and P3 (10.0 ± 0.6, 10.3 ± 0.7, and 21.7 ± 1.3 g L^−1^) due to the culture medium composition (containing 10% seawater for P1 and P2 and 50% seawater for P3). The former differed significantly from all full-seawater conditions (*p* < 0.05; Dunn’s post hoc test). From phase P4 onwards (excluding the 16-day progressive salt acclimation period), when the culture medium contained full seawater, the mean salinity was 37.9 ± 2.2 g L^−1^, ranging from 28.0 (measured in P6NS) to 49.7 g L^−1^ (measured in P4). Salinity in P6NS (36.3 ± 3.0 g L^−1^) remained slightly lower than in the other full-seawater phases (*p* < 0.05; Dunn’s post hoc test) due to the initial intentional reduction in salinity concentration aimed at reducing the synergistic stress effect of light and salinity on the culture (see also below).

Pure CO_2_ was supplied on demand at flow rates ranging from 0.1 to 1 L min^−1^ during phases P1, P2, and P3 to maintain pH below 10.5. In contrast, no CO_2_ supply was required in full-seawater cultures to prevent pH increase above this threshold. The only exception was phase P7.CO_2_^+^, where CO_2_ was intentionally supplied at a fixed rate (0.1 L min^−1^) to test its effect on productivity and biomass composition (Table 1). Overall, among the CO_2_^−^ supplemented phases, the average CO_2_ supply rates in P1 and P2 were significantly higher than those in P3 and P7.CO_2_^+^, and they also differed from each other, whereas P3 and P7.CO_2_^+^ did not differ significantly (*p* < 0.05; Dunn’s post hoc test).

ER across the experimental phases averaged 2.83 ± 1.49% day^−1^. A decreasing trend in ER was observed with the increase in culture salinity under the shaded conditions (*r* = −0.417; *p* < 0.001), with P1 and P2 exhibiting the highest ER (3.7 ± 1.5 and 3.5 ± 1.6%, respectively; *p* < 0.05; Dunn’s post hoc test; Table 1). P6_Total_ exhibited one of the lowest ER rates (2.4 ± 1.2% day^−1^). Notably, P6NS (non-shaded phase) did not differ significantly from the ER observed in phases P1, P2, P3, P5, and P7.CO_2_^−^ (*p* > 0.05; Dunn’s post hoc test).

Overall average Cx Start-to-End concentrations ranged from 0.80 ± 0.23 to 1.02 ± 0.23 gDW L^−1^ on a dry weight basis, with a minimum of 0.19 gDW L^−1^ for Cx Start and a maximum of 1.46 gDW L^−1^ for Cx End. The phase with the lowest average Cx Start was P1 (0.54 ± 0.12 gDW L^−1^), whereas the phase with the highest one was P2 (0.94 ± 0.33 gDW L^−1^) (Table 1). The average difference between Cx End and Cx Start across the experimental phases ranged from 0.16 (P4) to 0.26 (P6_Total_) gDW L^−1^.

**Figure 1:**
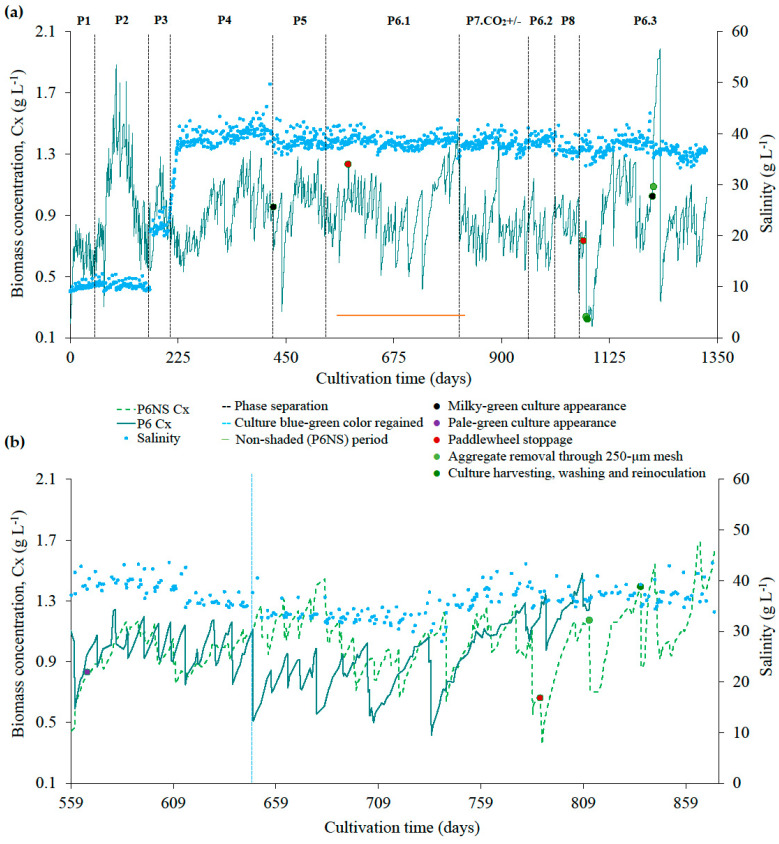
(**a**) Biomass concentration (Cx, gDW L^−1^; solid dark green line) and salinity (blue dots) of the *L. platensis* BEA 1257B culture during the semi-continuous cultivation in the 10,000 L RW inside greenhouse during the experimental phases P1 (day 0–50), P2 (day 50–166), P3 (day 166–207), P4 (day 207–424), P5 (day 424–539), P6.1 (day 539–813), P7.CO_2_^+^ (day 813–868), P7.CO_2_^−^ (day 868–939), P6.2 (day 939–1013), P8 (day 1013–1061), P6.3 (day 1061–1328). Vertical dashed lines indicate the end of each phase and the beginning of the next one. Orange line indicates the period of the non-shaded assay (P6NS, see below (**b**)). (**b**) Biomass concentration (Cx, gDW L^−1^; dashed light green line) and salinity (blue dots) of the *L. platensis* BEA 1257B culture over time during semi-continuous cultivation in a 10,000 L RW inside greenhouse without shading (559–872). The dark green line corresponds to Cx under the same culture conditions in the adjacent shaded culture (P6) (day 559–812). Purple point indicates when culture turned pale-green in appearance, blue vertical line marks the point at which the culture regained its characteristic blue-green color (Day 643) after appearing pale-green. For both graphics, red points indicate times in which paddle wheels were stopped due to electro-mechanical failures, orange points indicate times in which culture was filtered through a 250 µm mesh to remove biomass aggregates, black points indicate when culture turned milky green in appearance, and green points indicate harvesting, washing, and re-inoculation of the biomass.

Figure 1a presents biomass concentration (Cx) and salinity across experimental phases P1–P8 during the 1328-day semicontinuous cultivation of *L. platensis*. A focused comparison between the P6 phase under shaded (P6.1) and non-shaded conditions (P6NS) within the same temporal framework (7 September 2022 to 19 May 2023, days 559 to 812) is provided in Figure 1b and Table 2. Temperatures in the cultures ranged from 14.9 to 30.7 °C, with no significant difference between conditions (mean P6: 21.2 ± 2.5 °C; mean P6NS: 21.6 ± 3.0 °C; *p* > 0.05; *t*-test), suggesting that shading did not considerably alter culture thermal conditions under greenhouse conditions, at least based on measurements predominantly taken during the morning. Slightly higher pH values were found in the non-shaded compared to the shaded culture (9.53 ± 0.4 vs. 9.23 ± 0.4, *p* < 0.05; *t*-test). ER was also significantly higher in the non-shaded culture (3.2 ± 1.0% day^−1^, max. 6.1% day^−1^) compared to the shaded one (2.1 ± 0.9% day^−1^, max. 3.7% day^−1^; *p* < 0.05; *t*-test), pointing out the protective role of shading in minimizing water loss.

Salinity in the non-shaded culture ranged from 28.0 to 43.6 g L^−1^, with an average of 36.0 ± 3.2 g L^−1^. These values were significantly lower than in the shaded culture (38.5 ± 1.2 g L^−1^; *p* < 0.05; *t*-test), due to specific stress-mitigation strategies applied during the early stages of cultivation in P6NS. Indeed, at the beginning of the cultivation period, the non-shaded culture displayed signs of pigment degradation, with cells appearing pale-green (purple point on Figure 1b, day 566), possibly due to synergistic effects of intense solar exposure and high salinity, as previously described for the shaded culture when the shading screen was temporarily removed. In an attempt to reduce this stress, salinity levels were intentionally reduced by diluting the culture with freshwater after harvesting. Then, Cx Start in the following semicontinuous cycles was progressively increased. Once the culture showed signs of recovery, salinity was gradually increased by compensating evaporation with seawater, and Cx Start was moderately reduced (Figure 1b). Still, average Cx Start in P6NS was similar to P6 (0.91 ± 0.19 vs. 0.86 ± 0.21 gDW L^−1^, respectively; *p* > 0.05; *t*-test) and both systems ultimately reached quite comparable mean final concentrations (Cx End: 1.09 ± 0.18 vs. 1.10 ± 0.17 gDW L^−1^, P6NS and P6, respectively).

**Figure A2:**
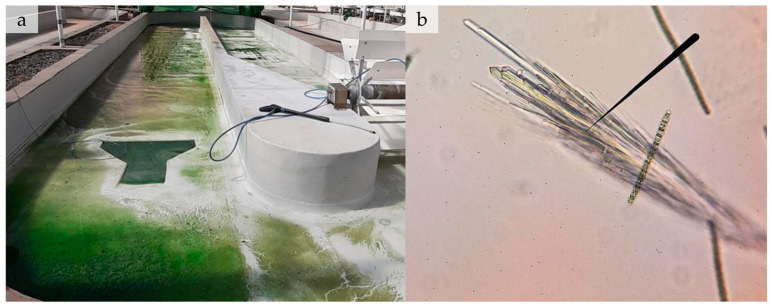
Outdoor cultures of *L. platensis* BEA 1257B in full-seawater under a greenhouse in 80 m^2^ RW. The images show appearance of salt precipitates in the culture: (**a**) at the bottom of the RW and (**b**) under light microscopy (200× magnification).

The shaded culture experienced a few episodes of milky green appearance (Appendix A.1, Figure A1), most likely due to the formation of precipitates (*n* = 2; Appendix A.2, Figure A2). When this first occurred, in the P4 phase (black point in Figure 1a, day 424), the successful strategy to restore the blue-green natural appearance consisted of transferring part of the culture into a clean RW and reducing carbonate salts by 40% (starting phase P5). Successively, both P6 and P6NS cultures experienced disruptions due to electro-mechanical failures that halted the paddle wheels (marked with a red point in Figure 1a,b), resulting in a complete loss of agitation for several hours. This occasional absence of mixing led to a quick natural flocculation of the cells at the surface, which was not effectively dispersed after returning to normal stirring conditions. To manage this operational issue in the shaded culture (Figure 1a, red point on day 579), biomass aggregates were removed with a manual net, then a portion of the culture was transferred into a clean RW. On the other hand, this strategy was not sufficient to recover the non-shaded culture, which remained stagnant and milky green for a longer period (Figure 1b, red point on day 788), and where salt precipitation occurred. Therefore, the entire culture was passed through a 250 µm mesh to remove larger precipitates (Figure 1b, orange point on day 812). As the pale-green milky appearance persisted, it was fully harvested on a vibrating filter, and the biomass was washed with seawater before being re-inoculated into a new RW with a fresh medium (Figure 1b, green point on day 845). This approach proved effective, allowing rapid recovery and growth. Consequently, when the next paddle wheel failure occurred in the shaded culture (Figure 1a, red point on day 1072), the same filtering, harvesting, and washing procedure was applied (Figure 1a, orange and green points on day 1215). The higher variation in biomass concentration observed afterwards (Figure 1a) was due to the temporary transfer of the culture to a 1600 L RW for a few days, while the original RW was cleaned. During the last milky green episode, in the P6.3 phase (black point on Figure 1a, day 1211), the same method was applied again, except for the 250 µm filtration step, as no biomass aggregates were present (green point in Figure 1a, day 1216).

Throughout the daily microscopic observations, rotifers were sporadically seen in *L. platensis* culture; ciliates belonging to the genus *Euplotes* were detected at low abundances when the culture had a low pH (<9.2). No evidence of grazing behavior on *Limnospira* cells was observed for either. Regarding the presence of photoautotrophic organisms, unidentified diatoms were occasionally observed at a relative abundance of <3%.

### 2.2. Productivity Trends

The average culture volumetric productivity on a dry weight basis throughout the experimental phases ranged between 0.029 ± 0.011 gDW L^−1^ day^−1^ in P4 and 0.075 ± 0.025 gDW L^−1^ day^−1^ in P2 (Table 3). Lower productivities were generally observed in the phases with the full-seawater medium (P4 onwards, mean < 0.05 gDW L^−1^ day^−1^ for all) in comparison to the phases with 10% of seawater (P1 and P2, >0.05 gDW L^−1^ day^−1^ for both), with statistically significant differences observed between P1 and P2 vs. P4, P6, and P7.CO_2_^−^ (*p* < 0.05; Dunn’s post hoc test). A comparison of productivity between the shaded P6 (0.031 ± 0.011 gDW L^−1^ day^−1^) and non-shaded P6NS phase (0.031 ± 0.019 gDW L^−1^ day^−1^) in the same temporal framework did not reveal significant differences (*p* > 0.05; *t*-test). When considering the long-term volumetric productivity of the culture in full seawater under optimized conditions (P6_Total_, 615 days), a mean value of 0.033 ± 0.011 gDW L^−1^ day^−1^, corresponding to an areal productivity of 4.1 ± 1.4 gDW m^−2^ day^−1^, was obtained by recirculating the culture medium. Extrapolating this areal productivity value to year-round operation per hectare of culture surface (360 days × 10,000 m^2^) results in an estimated annual biomass productivity of 14.8 ± 5.0 tons ha^−1^ year^−1^ for *L. platensis* in the tested system.

The independent variables corrected G_0_, medium recirculation condition (yes/no), salinity, concentration of KNO_3_, NH_4_H_2_PO_4_, and total carbonates (CO_3_), CO_2_ supply rate, and initial biomass concentration (Cx Start) were included in the adjusted GLM to test their effect on *L. platensis* productivity. When all cultivation phases were included in the GLM, productivity showed a negative significant association with salinity and a positive significant association with phosphate concentration, respectively (*p* < 0.05). However, when data from the P6NS condition (non-shaded cultivation) were excluded from the model, G_0_ became the only variable significantly positively related to productivity (*p* < 0.001).

### 2.3. Biomass Composition Comparison

#### 2.3.1. Proximal Composition and C-Phycocyanin Content

**Figure 2:**
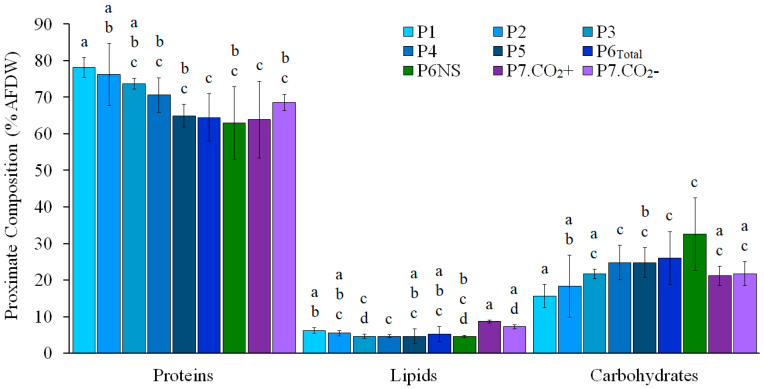
Protein, lipid, and carbohydrate content of *L. platensis* BEA 1257B biomass (expressed in % of ash-free dry weight, AFDW), across the different cultivation phases P1–P7. For each parameter, letters above the bars indicate statistically significant differences between phases among the recorded cycles (*p* < 0.05) based on Dunn’s post hoc test. Data represent the mean values for each cultivation phase, with error bars showing the corresponding standard deviation.

Ash- and water-free protein content ranged from 48.0 to 84.2%, with an overall average of 70.3 ± 7.9% AFDW (Figure 2). A significant difference in protein levels (≃17% lower) was observed between P1 and P2, which showed the highest protein contents (78.1 ± 2.8 and 76.2 ± 8.5% AFDW, respectively), and P6_Total_ and P7.CO_2_^+^, which showed the lowest protein contents (64.4 ± 6.5 and 63.9 ± 10.5% AFDW; *p* < 0.05; Dunn’s post hoc test). Moreover, the non-shaded phase P6NS (62.9 ± 9.9% AFDW) did not show significant differences compared to the other seawater-based phases (P4, P5, P6_Total_, and P7; *p* < 0.05; Dunn’s post hoc test). A decreasing trend in protein content with increasing in culture salinity and decreasing nitrogen availability is suggested. However, while protein content was strongly negatively correlated with salinity (*r* = −0.60, *p* < 0.01), it showed only a weak positive correlation with KNO_3_ concentration in the make-up medium (*r* = 0.33; *p* < 0.001).

Lipid content ranged from 2.1 to 8.9% AFDW, with an overall mean of 5.4 ± 1.3% AFDW (Figure 2). A slight decreasing trend in lipid content was observed with increasing salinity, except for P7.CO_2_^+^ (8.7 ± 0.4% AFDW) and P7.CO_2_^−^ (7.2 ± 0.6% AFDW), both of which exhibited the highest lipid values detected. Nevertheless, the lipid content of P1 (6.1 ± 0.8% AFDW) and P2 (5.5 ± 0.7% AFDW) was not significantly different from the optimized full-seawater phase P6_Total_ (5.2 ± 2.0% AFDW; *p* < 0.05; Dunn’s post hoc test). No significant correlation was observed between lipid content and any of the tested variables (*p* > 0.05).

Carbohydrate content ranged from 10.0 to 40.2% AFDW, with an overall mean of 22.4 ± 7.2% AFDW, being generally higher in full-seawater phases (P4, P6_Total_, and P6NS) than 10% seawater phases (P1, P2; *p* < 0.05; Dunn’s post hoc test; Figure 2). Actually, carbohydrate content exhibited a significant moderate positive correlation with salinity (*r* = 0.57; *p* < 0.001).

**Figure 3:**
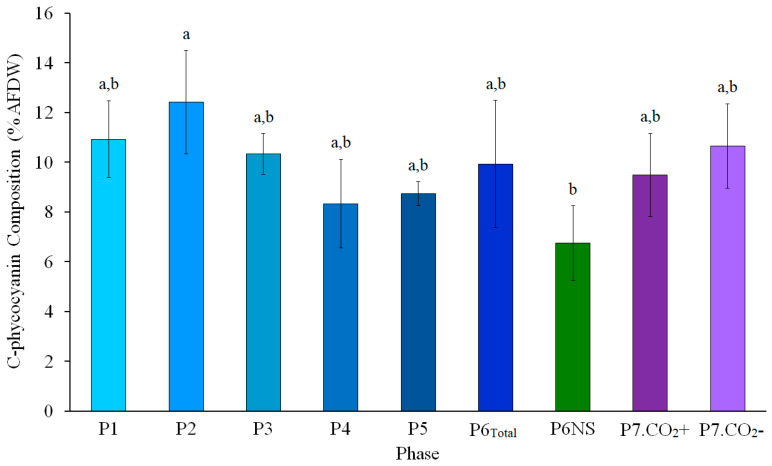
C-phycocyanin content (% of ash-free dry weight, AFDW) of *L. platensis* BEA 1257B biomass across the different cultivation phases (P1–P7). Letters above the bars indicate statistically significant differences between phases among the recorded cycles based on Tukey’s post hoc test (*p* < 0.05). Data are presented as mean values for each phase, with error bars representing the corresponding standard deviation.

The concentration of C-phycocyanin (C-PC) along the three years of cultivation ranged from 3.7% to 15.5% AFDW, with an average of 9.5 ± 2.4% AFDW. C-PC content showed a moderate negative correlation with salinity (*r* = −0.40; *p* < 0.01). Significant differences were observed between *L. platensis* biomass cultivated at 10% seawater (P2: 12.4 ± 2.1% AFDW), where the highest C-PC content was observed, in comparison with full seawater under non-shaded conditions year-round (P6NS: 6.7 ± 1.5% AFDW). Within the same temporal framework, C-PC content was significantly higher in P6.1 (9.8 ± 3.1% AFDW) than in P6NS (6.7 ± 1.5% AFDW; *p* < 0.001; *t*-test). P6_Total_, which represents the most optimized stage of the cultivation process in full seawater, showed a C-PC content (9.9 ± 2.5% AFDW) not significantly different from P1 and P2 (Figure 3). Delving deeper into the color appearance differences between P6 and P6NS, the separation of relevant monitored parameters according to the pigmentation state associated with P6NS (green–pale vs. dark green–blue) revealed clear differences (Table 4). During the green–pale stage, P6NS showed significantly lower C-PC content (6.5 ± 0.5% AFDW), productivity (0.024 ± 0.015 gDW L^−1^ day^−1^), and Fv/Fm (0.428 ± 0.042) compared with shaded P6 cultures recorded within the same period (10.7 ± 2.2% AFDW; 0.037 ± 0.010 gDW L^−1^ day^−1^; and 0.524 ± 0.073, respectively). Nevertheless, the controlled ~10% reduction in culture salinity led to a shift toward a dark green–blue pigmentation state, accompanied by higher Fv/Fm values and C-PC content under non-shaded operation (Table 4).

When assessing the effect of two different drying methodologies on the biomass, a two-way ANOVA revealed significant differences in protein content associated with both the batch factor and the processing method (*p* < 0.01 for both). Lyophilized samples exhibited a significantly higher protein content with respect to spray-dried samples (82.6 ± 0.6 vs. 81.0 ± 1.0% AFDW); however, the difference was less than 2%. Lipid and carbohydrate content showed significant differences associated with the batch factor. Nevertheless, while no significant differences in the former were observed between processing methods (6.2 ± 0.2 and 5.7 ± 0.8% AFDW for lyophilized and spray-dried samples, respectively), higher carbohydrate contents were observed in spray-dried samples compared with lyophilized samples (13.7 ± 0.2 and 11.2 ± 0.1% AFDW, respectively). On the other hand, no significant differences were observed for phycocyanin content, neither for batch nor for processing method factor (C-PC overall mean: 10.1 ± 2.0% AFDW).

Gamma linear model (GLM) analyses were conducted to evaluate whether specific cultivation and process parameters could significantly affect the biochemical composition of *L. platensis* biomass. The model included the following explanatory variables: corrected G_0_, medium recirculation (yes/no), salinity, concentrations of KNO_3_, NH_4_H_2_PO_4_, and total carbonates (CO_3_), CO_2_ supply rate, and initial biomass concentration (Cx Start). Additionally, based on the differences observed in the previous compositional comparison, the biomass processing method—freezing, lyophilization, and spray-drying—was included in the model. None of these variables showed statistically significant associations with protein, carbohydrate, or phycocyanin content. However, in both cases, the GLM identified a positive association between lipid content and frozen biomass processing method (*p* < 0.001).

The ash content of *L. platensis* BEA 1257B biomass across the different cultivation phases is shown in Table 5. Overall, ash content varied from 4.0 to 28.3% DW, with an overall average value of 13.4 ± 4.7%. Phase P5 exhibited the lowest ash content (7.9 ± 5.1% DW), while P7.CO_2_^+^ showed the highest (17.6 ± 9.2% DW). No statistical differences were observed between phases.

**Figure 4:**
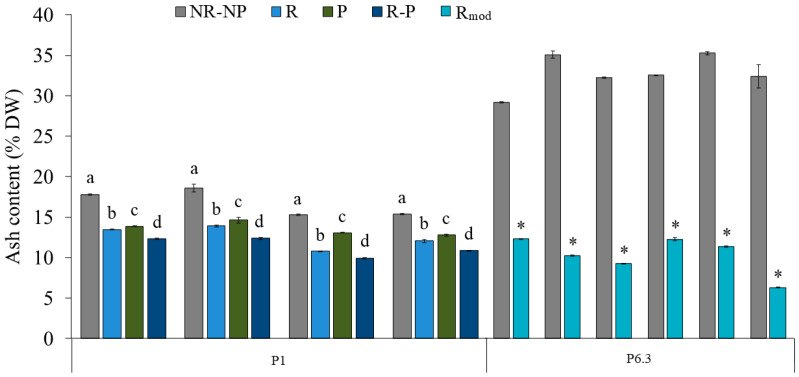
Ash content (% DW) in *L. platensis* BEA 1257B fresh biomass (slurry) harvested using the vibrating filter as it is (neither rinsed nor pressed, NR-NP) and after the subsequent post-harvesting steps of washing and/or dewatering: Rinsing with freshwater (5:1 *v/v* FW) directly onto the vibrating filter (R); Manually pressing through a 30 µm nylon net (P); Rinsing with freshwater (5:1 *v/v* FW) and manually pressing through a 30 µm nylon net (R-P); Rinsing with freshwater (5:1 *v/v* FW), filter was turned off after 1.5 min from the end of biomass rinsing (R_mod_). ^a,b,c,d^ Letters denote statistically significant differences between experimental conditions among the recorded cycles in P1 (*p* < 0.05; Tukey test). * Denote significant differences between experimental conditions in P6 (*p* < 0.05; *t*-test).

It has to be considered that all the harvest batches of fresh biomass were rinsed with freshwater directly on the vibrating filter (5:1 *v/v* FW; see also Section 4.3), specifically with the aim of reducing the ash content in the biomass due to the presence of residual salts from the culture medium. This post-harvesting step was chosen based on a comparison of ash content in four harvest batches of fresh biomass, each subjected to different washing and dewatering procedures at the beginning of phase P1 (Figure 4). Freshwater rinsing alone (R) resulted in a significant 25.2 ± 3.2% reduction in ash content on a dry weight basis with respect to the non-rinsed and non-pressed fresh biomass (NR-NP, control). Rinsing alone was more effective than pressing alone (P: 18.7 ± 3.6% reduction with respect to NR-NP) in reducing ash content for all the tested samples and was considerably less time-consuming. When both processes were applied in combination (R-P), the highest reduction was achieved (32.2 ± 2.6%). However, the final average ash content of 12.5 ± 1.3% DW obtained using the rinsing step, starting from the initial 16.8 ± 1.7% DW in the NR-NP slurry, was considered satisfactory for food market purposes. Therefore, pressing was not further implemented during the subsequent phases, prioritizing a more operationally efficient approach, particularly in relation to subsequent spray-drying of the biomass, where low viscosity is required.

Ash content in R biomass revealed a progressive, although not significant, increase in both amount and variability as seawater concentration in the culture medium increased (average ash content in P4–P7: 14.1 ± 4.8% DW). Hence, during the last phase of cultivation (P6.3), the rinsing step was slightly modified by leaving the biomass on the operating vibrating filter for an additional 1.5 min after the end of the biomass rinsing, allowing further dewatering of the slurry (R_mod_; see also Section 4.3). The comparison across six harvest batches of fresh biomass revealed that ash content in the slurry was reduced by 68.5 ± 7.9% on average, from 34.0 ± 2.2% DW in NR-NP to 10.7 ± 2.3% DW in R_mod_, with the latter being not significantly different from the final average ash content of 12.5 ± 1.3% DW obtained in P1 using the R step.

#### 2.3.2. Mineral Composition and Microbiological Quality

Mineral composition, trace element, and heavy metal content of *L. platensis* biomass across the cultivation phases P1, P3, P4, and P6_Total_ is shown in Table 6. K content ranged from 910.1 ± 124.1 mg 100 g^−1^ DW in P3 to 1645.4 ± 568.7 mg 100 g^−1^ DW in P6_Total_. K was the most abundant mineral in the biomass in P1 (1359.5 ± 395.4 mg 100 g^−1^ DW), the phase with the lowest volume of seawater (10%) but the highest concentration of KNO_3_ in the culture medium (2 g L^−1^). On the other hand, K content was significantly lower in P3, the phase with 50% seawater and 1 g L^−1^ of KNO_3_ in the culture medium, in comparison with the other phases.

Na levels ranged from 375.0 ± 97.7 mg 100 g^−1^ DW in P3 to 2822.2 ± 2460.4 mg 100 g^−1^ DW in P6_Total_. As expected, the highest contents of Na were detected in the biomass grown in full seawater (P4 and P6_Total_), whereas the lowest were observed at 10 and 50% seawater (P1 and P3, respectively). Na levels in P1 did not differ significantly from those in P4, possibly due to the higher NaHCO_3_ content (8 g L^−1^) in the P1 culture medium. Mg and Ca showed a similar pattern, both being approximately four-fold higher in the biomass grown in full seawater (P4 and P6_Total_) with respect to that grown in 10% seawater (P1). High Mg and Ca contents (although not significantly with respect to P4 and P6_Total_) were observed in the biomass grown in 50% seawater (P3: 1238.4 ± 92.7 mg 100 g^−1^ DW for Mg and 530.3 ± 223.9 mg 100 g^−1^ DW for Ca), possibly due to partial precipitation of these salts in the culture medium together with CO_3_ at high alkalinity.

Among trace metals, Fe was the most abundant in all phases (range: 27.2 ± 7.2 to 58.1 ± 21.2 mg 100 g^−1^ DW, in P4 and P1, respectively), with lower but not significantly different levels detected in the biomass grown in full seawater with respect to the biomass grown at 10 and 50% seawater. On the other hand, Mn, Zn, and B (range: 0.6–4.7 mg 100 g^−1^ DW) were all approximately five-fold higher in P6_Total_ with respect to P1, although the difference was not significant for B. Cu levels were below 1 mg 100 g^−1^ DW across all phases, with a significant decreasing trend with increasing culture salinity.

Heavy metals were all below 100 mg kg^−1^ DW on average in the phases P1, P3, P4, and P6_Total_. The only exception was As, which reached a mean concentration of 230 ± 172 × 10^−3^ mg kg^−1^ DW in the optimized full-seawater conditions (P6_Total_) while being not detected in 10% and 50% seawater (P1 and P3, respectively). Cr and Pb were detected at all phases, with higher values in P6_Total_ with respect to P1, although the difference was not significant for Cr. Se was detected in the culture carried out at a higher % of seawater (P3 onwards), whereas Mo was only detected at the lowest % of seawater (P1); however, the concentrations of these metals were on average ≤ 5 × 10^−3^ mg kg^−1^ DW. Cd was significantly higher in P6_Total_ as compared with P1 (*p* < 0.05; *t*-test), and it was not detected in P3 and P4. Neither mercury (Hg) nor cobalt (Co) was detected in any phase.

The microbiological profile of the *L. platensis* biomass was assessed across the different cultivation phases except for P5 (Table 7). The total aerobic mesophilic flora counts (overall average: 7.0 ± 8.6 × 10^3^ cfu g^−1^) ranged from 1.0 × 10^2^ cfu g^−1^ to 2.1 × 10^4^ cfu g^−1^ (P1 to P7.CO_2_^+^). Significant differences were only observed between phases P6_Total_ and P6NS (*p* < 0.05; Dunn’s post hoc test), whereas the latter did not differ from the other phases. Yeasts and molds were below the detection limit of the analytical method except in four samples, where they were close to the detection limit. The assayed bacterial pathogens (i.e., Enterobacteriaceae, total coliforms, *Escherichia coli*, *Staphylococcus* spp., *Clostridium perfringens,* and *Salmonella* spp.) were either below the detection limit of the analytical method or absent.

#### 2.3.3. Composition Profile of Spray-Dried *L. platensis* Biomass Cultivated in Optimized Full-Seawater Conditions

*L. platensis* biomass cultivated under optimized full-seawater conditions (P6_Total_) and subjected to spray drying showed a consistent nutritional profile (Table 8). Results in terms of macrocomponents highlighted an average protein, lipid, and carbohydrate content of 46.4 ± 4.2, 17.2 ± 7.3, and 3.4 ± 1.4 g 100 g^−1^, respectively. Sugar and dietary fiber contents were 2.3 ± 0.1 and 7.1 ± 4.0 g 100 g^−1^, respectively. The phycocyanin content averaged 9.64 ± 2.69 g 100 g^−1^ of the total biomass. Complementary analyses of the amino acid profile highlighted high levels of glutamate (16.62 ± 1.16 g 100 g^−1^ protein), followed by aspartate (9.33 ± 1.53), leucine (9.09 ± 1.45), alanine (7.88 ± 1.19), and valine (6.59 ± 0.74). The fatty acid (FA) profile was marked by high proportions of palmitic acid (PA: 42.9 ± 0.3%), linoleic acid (LA: 21.0 ± 1.5%), and γ-linolenic acid (GLA: 20.7 ± 1.3%), together accounting for almost 85% of the total FA. α-linolenic (ALA), arachidonic (ARA), eicosapentaenoic acid (EPA), and docosahexaenoic acid (DHA) were either sporadically detected in negligible amounts or absent. Vitamin content included thiamine (B1): 1.04 mg kg^−1^, riboflavin (B2): 39.7 mg kg^−1^, niacin (B3): 11.5 mg kg^−1^, biotin (B8): 2.6 µg 100 g^−1^, and cyanocobalamin (B12): 4.6 µg 100 g^−1^. In addition, further analysis revealed a maximum iodine and nickel content of 2.2 and 2.8 mg kg^−1^, respectively. *L. platensis* BEA 1257B, cultivated under optimized full-seawater conditions, tested negative for PAHs, pesticides, and mycotoxins. Potential allergen contamination associated with seawater cultivation was excluded based on the absence of detectable crustacean, fish, and mollusk DNA.

### 2.4. Energy, Freshwater, Effluents, and Nutrients Savings

**Figure 5:**
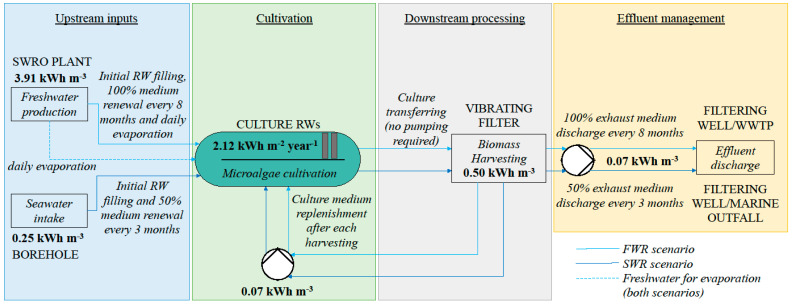
Simplified flow diagram of the optimized process of *L. platensis* cultivation in optimized full-seawater conditions with culture medium recycling developed in this study (SWR, dark-blue solid lines) and the well-established long-term cultivation process of *L. platensis* in freshwater with culture medium recycling (FWR, light-blue solid lines; [3,27,35]). Main process stages are underlined and differentiated by using colored blocks. Process steps are in italic. Electric consumption of each process step is marked in bold. Main infrastructure and equipment involved in the process are detailed in capital letters. WWTP: Wastewater Treatment Plant.

Energy, freshwater, and fertilizer savings achieved through culture medium recirculation strategy under optimized full-seawater conditions (SWR), expressed relative to conventional freshwater cultivation (FWR; Figure 5), are shown in Table 9 (see also Section 4.6 for a detailed description of assumptions). Results are presented both on a surface-based basis and normalized per unit of biomass, protein, and phycocyanin produced.

On a surface-based basis (10-ha facility), SWR reduced energy consumption along the whole cultivation process by 41.9 MWh year^−1^ (10.5%) and freshwater use by 12,000 m^3^ year^−1^ (12%) compared with FWR. Fertilizer savings in the make-up medium amounted to 14.0 t year^−1^ for KNO_3_ and 0.12 t year^−1^ for NH_4_H_2_PO_4_, corresponding to reductions of 58.3% and 16.7%, respectively. Total carbonate salts (CO_3_, supplied as Na_2_CO_3_ and NaHCO_3_), NaCl, and MgSO_4_·7H_2_O were reduced by 23.2 t year^−1^ (67.7%), 60.0 t year^−1^ (100%), and 1.9 t year^−1^ (100%), respectively.

When results were normalized per unit of product, a trade-off between productivity and resource efficiency became evident. Negative values were mainly observed when normalization was based on protein production, with reductions of −85.1% for energy and −74.4% for freshwater, reflecting the combined effect of lower biomass productivity and reduced protein content under SWR conditions. In contrast, normalization based on phycocyanin production yielded consistently positive savings, with energy and freshwater reductions of 6.6% and 12.0%, respectively, and approximately one-third reductions for both KNO_3_ and NH_4_H_2_PO_4_. For biomass-based normalization, intermediate results were observed, with moderate reductions in some inputs and limited increases in others. Despite this variability, substantial savings in specific inputs, such as carbonate salts (up to 67.7%) and NaCl (100%), were maintained across all functional units (Table 9). MgSO_4_·7H_2_O was additionally saved at a rate of 0.03 t per ton of dried biomass harvested since this Mg salt is not replenished in SWR, in contrast to FWR, where 30 g are reintegrated in the culture per kg of dry biomass.

When considering the number of major inorganic nutrients and ions of environmental concern prevented from being discharged into the environment via effluents (Table 10), SWR consistently outperformed FWR. On a surface-based basis, reductions of 1.94 t year^−1^ (89.6%) for N and 0.2 t year^−1^ (86.1%) for P were achieved, corresponding to nine- and eight-fold lower N and P discharges, respectively. These reductions remained high when expressed per unit of biomass, protein, and phycocyanin produced, with values ranging from 79.3% to 96.5% for N and from 72.5% to 86.1% for P, while the chloride (Cl^−^) reduction remained complete (100%) across all functional units, indicating that the environmental benefits of SWR are maintained regardless of the functional unit considered.

According to the results obtained, SWR would generate an effluent with <50 mg L^−1^ of nitrate and <5 mg L^−1^ of phosphate (Appendix A.3, Table A1), values compatible with regulatory discharge limits. Additionally, assuming negligible Cl^−^ assimilation by *L. platensis*, 36.4 t year^−1^ of Cl^−^ are introduced as NaCl from an external source and discharged into the environment in FWR, whereas in SWR, all Cl^−^ originates from the seawater itself and is returned to the marine environment without net external input (Table 10).

## 3. Discussion

The whole experimental cultivation period included in this study spanned 1328 days. Moreover, the culture operating in semicontinuous mode is still ongoing at the time of submitting this manuscript for publication. To the best of our knowledge, this represents the longest-term outdoor culture optimization study at a pilot scale of *L. platensis* reported to date. Notably, it is also the longest conducted in a seawater-based medium (1105 days), of which 615 days included culture medium recirculation and reduced nutrient supply. Unlike other cultivation studies on seawater-based *Limnospira* [12,23] and other marine species that lasted up to 1 year [31,36], this full-seawater *L. platensis* culture has been successfully operating for 4 years as of this writing.

### 3.1. Full Seawater Culture Acclimation and Optimization

In this study, *L. platensis* BEA 1257B was first acclimated indoors using a medium containing 10% *v/v* natural seawater (final salinity 3.7 g L^−1^). Acclimation to full seawater (37 g L^−1^ salinity) was subsequently performed directly outdoors. During phase P3, salinity was increased in a single step from 10% to 50% seawater (18.5 g L^−1^), whereas at the beginning of phase P4, salinity was gradually raised by compensating daily evaporation with seawater. This progressive acclimation strategy applied to physiologically mature outdoor culture in P4, successfully tested and reported for the first time by Alemán et al. [37], represents one of the main findings of this study. Prior to this outdoor trial, several attempts were made at the ITC to acclimate the *L. platensis* Canarian strain to seawater under controlled growth chamber conditions using a conventional stepwise salinity increase method [21]. The strain adapted readily up to 50% seawater medium; however, growth was strongly inhibited at higher seawater concentrations, and complete culture collapse occurred above 75% seawater. Gradual outdoor acclimation of other *Limnospira* strains has also been recently reported under comparable pilot-scale, outdoor conditions in raceway systems [12,24]. This outcome could potentially be related to the greater metabolic plasticity that microalgae may exhibit under natural environmental conditions compared to controlled indoor cultivation [38,39,40].

Nutrient optimization assays during the long-term cultivation (phases P2, P5, P6, and P7) were used to (i) generate an effluent that complies with the local water discharge regulations, even when untreated prior to discharge; (ii) minimize salt precipitation in the culture medium; and (iii) reduce the production costs while maintaining consistent biomass productivity and quality for the food and feed sector. Although the reduction in N and P supply may have partially affected protein content and productivity under the optimized full-seawater conditions (P6_Total_; see Section 3.2 and Section 3.3), it is important to note that this optimization resulted in residual nitrate and phosphate concentrations in the effluent after each harvest of <50 mg L^−1^ and <5 mg L^−1^, respectively (Appendix A.3, Table A1). These values are generally below the current legal limits for effluent discharge to the environment in the Canary Islands (10 mg L^−1^ for both N-NO_3_ and total P; [41]) and allow direct discharge to the sea through either a marine outfall or a filtering well. While the maximum renewal rates of the culture medium used for *Limnospira* cultivation in industrial and pilot-scale facilities are well documented [3,24,27], residual nutrient concentrations in *Limnospira* cultivation effluents and effluent disposal strategies are rarely reported [26]. A recent study by Pistocchi et al. [27] revealed residual N–NO_3_ and P–PO_4_ concentrations in the effluent of 181 and 20 mg L^−1^, respectively, which are approximately nine-fold and two-fold higher than the local discharge limits. It should also be noted that, in addition to the high nitrate and phosphate loads, effluents from conventional *Limnospira* culture media exhibit high alkalinity and sodium content (due to the use of up to 16 g L^−1^ NaHCO_3_ and/or Na_2_CO_3_ and up to 4 g L^−1^ NaCl; [3,27,42,43,44]). These chemical properties strongly limit the possibility of directing the effluent to a municipal wastewater treatment plant or using it for irrigation without prior extensive dilution. In a recent study on *L. maxima* cultivated in full seawater in Saudi Arabia, the effluents were discarded in evaporation ponds [24], raising concerns about the management of the resulting solid residues.

It should be considered that the reduction in phosphate applied in this study was not only aimed at lowering its residual concentration in the final effluent but also at minimizing the precipitation of inorganic salts (mainly carbonates, calcium, and magnesium) in the culture medium occurring at high alkalinity [45,46]. This phenomenon is common in conventional *Limnospira* cultures and can lead to a decrease in alkalinity, as well as a reduction in bioavailable iron and phosphorus in the system [3,44,47]. This issue becomes even more relevant in seawater cultures, due to the high Ca and Mg content, and can severely affect both culture performance and biomass quality. A fine-tuned balance of carbonate, bicarbonate, phosphate, and pH is required to prevent massive salt precipitation and avoid preliminary chemical treatment of seawater to remove Ca and Mg, whose additional cost would make its utilization less attractive [13,14,19]. For this reason, a 40% reduction in NaHCO_3_ and Na_2_CO_3_ in the make-up medium (both from 1.0 to 0.6 g L^−1^) was implemented at the beginning of P5, immediately after an intense episode of culture discoloration and precipitate formation observed in P4 (see Section 4.2.3), following 201 days of operation with medium recycling under full seawater. A plausible explanation for this phenomenon is the accumulation of Na, Ca, and Mg due to the use of desalinated water to compensate for evaporation during the semicontinuous cycles. Assuming a daily evaporation rate (ER) of 2.7% (Table 1) and considering the average Na, Ca, and Mg content in the freshwater (see Section 4.2.3), an additional input of approximately 488 mg L^−1^ Na (90 × 201 × 270/10,000), 65 mg L^−1^ Ca (12 × 201 × 270/10,000), and 103 mg L^−1^ Mg (19 × 201 × 270/10,000) would be expected after 201 days of cultivation. Based on this observation, phosphate was reduced in P6 as a preventive measure against the increase in salinity and, consequently, in Ca and Mg ion concentrations.

Maintaining the pH below the common values used for conventional cultivation of *Limnospira* is also necessary to limit salt precipitation in seawater cultures of this cyanobacterium [14,23]. In this study, a progressive decrease in pH was observed with increasing salinity (*r* = −0.60; *p* < 0.05), as previously reported [15]. During the initial phases (P1–P3), when carbonate/bicarbonate availability and culture productivity were highest, and salinity was lowest, CO_2_ injection was required to prevent pH from exceeding inhibitory levels (>10.5), consistent with the patterns previously reported for high-alkalinity freshwater cultures of *Limnospira*, where intense photosynthetic activity rapidly consumes inorganic carbon [9]. However, under optimized full-seawater conditions in our cultures, the combined effects of reduced carbonate supply, lower photosynthetic carbon demand, and the natural buffering capacity of seawater, together allowed us to stabilize culture pH near 9.3 without external CO_2_ supply. The P7.CO_2_^+^ phase further indicated that supplemental CO_2_ did not produce measurable effects on biomass productivity or biochemical composition, demonstrating that CO_2_-based acidification is not required in SWR process, which could result in notable savings in *Limnospira* biomass production given the significant contribution of food-grade CO_2_ to overall production costs [48], as well as reduced CO_2_ wastage to the environment in raceway systems (up to 60%; [49]). These results contrast with previous seawater-based studies on *Limnospira* cultivation, which relied on active pH control through bicarbonate or citric acid additions, or continuous CO_2_ supplementation [12,24]. This difference may be related to the more efficient optimization of carbonate/bicarbonate use in our study and to the lower productivities (resulting in a lower C demand) observed. Productivity does not appear to be constrained by carbon or other nutrient limitations but rather by salinity and irradiance stress (see Section 3.2).

Throughout the whole experiment, biological contamination did not represent a significant issue for the culture. Both *Euplotes* sp. and rotifers appeared sporadically and were observed to come into physical contact with *L. platensis* cells, but not to actively graze them, as algal fragments were not detected inside their cell bodies under microscopic observation. Both heterotrophs promptly disappeared as pH increased above 9.2, possibly due to the inhibitory effects of highly alkaline conditions. Regarding *Euplotes*, this is consistent with previous observations in commercial freshwater cultures across China [50] and in a pilot culture of the same *L. platensis* strain under freshwater conditions [3]. In contrast, while rotifers were not detected by Guidi et al. [3], Yuan et al. [50] observed rotifers actively feeding on *Limnospira* cells, with *Brachionus plicatilis* identified as one of the most detrimental species to *Limnospira* cultures. However, the authors noted that the abundance and activity of *B. plicatilis* were highly dependent on the salinity of the cultures. It is therefore possible that the rotifers observed in our study belong to species that do not feed on *Limnospira*, or that their grazing activity was limited by the high salinity of full-seawater cultures. The addition of urea to the culture medium as a preventive measure against protozoan grazers may have contributed to limiting their appearance [26,51]. The strategy of maintaining the culture at high biomass concentrations throughout all optimization phases (0.80–1.02 gDW L^−1^), higher than those typically used in *Limnospira* cultivation (0.4–0.6 gDW L^−1^; [3,47,52]), although it could have partially limited the peak productivity (also see Section 3.2) served as a preventive measure to control biological contamination, which is recognized as a major economic challenge in mass cultivation of microalgae [53]. The same strategy was previously adopted by Guidi et al. [3] for the same strain cultivated in freshwater, in order to intensify the light self-shading and allelopathic effects on *C. sorokiniana* and diatoms [47]. While chlorophytes were not observed during the experiment, diatoms occasionally appeared at very low relative abundance (<3%). However, no significant contribution to the *L. platensis* BEA 1257B biomass composition is expected, as microscopic inspection revealed no diatom cells in the harvested biomass. This is likely due to their very low abundance in the culture and their small cell size, which prevents their retention on the filter during harvesting [44,47,52]. Taken together, these results confirm that *L. platensis* BEA 1257B is a consistent, contamination-resistant strain suitable for large-scale cultivation in the Canary Islands under the innovative technology proposed in this study, which constitutes a key prerequisite for successful microalgae production.

### 3.2. Key Drivers Affecting Culture Productivity

A limitation of the present study is that all cultivation phases were carried out sequentially in a single pilot-scale system over an extended period. Consequently, comparisons among phases represent temporal observations within the same culture rather than independent experimental replicates. As is typical for long-term outdoor cultivation studies, phase-to-phase differences may also reflect seasonal variability and long-term operational drift. For this reason, the statistical analyses are interpreted here primarily as descriptive and associative, and the identified relationships should be considered indicative patterns within the dataset rather than strictly causal effects. Given this premise, GLM analysis identified a negative association between productivity and salinity when all experimental phases were included in the model, whereas productivity was positively influenced by G_0_ when only shaded phases (all except P6NS) were considered. The dataset suggests that irradiance may interact with salinity to shape productivity patterns, with intense solar radiation and high salinity acting synergistically as environmental stressors under full-seawater conditions. This hypothesis is further supported by observed episodes of culture discoloration, after which the cultures recovered their original blue–green pigmentation either following shading or reduction in salinity. This discoloration is consistent with similar observations reported for *Limnospira* cultivated in full seawater and directly exposed to sunlight in outdoor raceway ponds [12,24], possibly as a consequence of increased production of reactive oxygen species (ROS) under high irradiance and UV exposure [53]. ROS can lead to the inactivation of photosystem II and disruption of the photosynthetic apparatus in *L. platensis* [54], resulting in reduced Fv/Fm values, decreased biomass productivity [12,15,55], and lower chlorophyll *a* and phycocyanin contents (see also Section 3.3), ultimately causing the pale-green appearance of the culture [21,56]. Conversely, the GLM analysis indicated a positive association between phosphate concentration—which was reduced from 0.06 g L^−1^ NH_4_H_2_PO_4_ in P1–P5 to 0.03 g L^−1^ from P6 onwards—and productivity, as previously observed for *Limnospira* [17,57].

Productivities on a dry weight basis recorded during the 10% seawater phases (P1 and P2: 0.054 ± 0.010 and 0.075 ± 0.025 gDW L^−1^ day^−1^, respectively) were within the same range as those reported by Guidi et al. [3] for the same *Limnospira* strain cultivated in the same raceway ponds using a conventional freshwater medium (0.060 ± 0.010 gDW L^−1^ day^−1^). The use of 10% seawater in medium preparation already resulted in a complete saving of calcium and magnesium inputs, in agreement with previous studies that have incorporated seawater as a source of micronutrients [58]. Supplementation of the freshwater culture medium with limited amounts of deep ocean water as a source of minerals and trace elements has also been implemented at an industrial scale, as exemplified by Cyanotech Corporation (Kailua-Kona, HI, USA), which commercializes Hawaiian Spirulina^®^.

In contrast, the average areal productivity of *L. platensis* BEA 1257B under optimized full-seawater conditions (P6_Total_: 4.1 ± 1.4 gDW m^−2^ day^−1^, equivalent to 14.8 ± 5.0 t ha^−1^ year^−1^) was approximately 30% lower than that reported for freshwater cultivation (6.0 ± 0.07 gDW m^−2^ day^−1^ [3]). It should be noted, however, that these latter values were obtained over a relatively short cultivation period and under higher nutrient supply, which would not comply with current effluent discharge regulations. A comparable decrease in productivity when shifting from freshwater to seawater media was also observed by Villaró et al. [12] in outdoor cultures of *L. platensis* in southern Spain, operated in similar raceway systems (80 m^2^ surface area, 0.13 m culture depth) inside a greenhouse.

The overall average productivity obtained here under optimized full-seawater conditions was lower than that reported by Villaró et al. [12] (16 ± 1.9 gDW m^−2^ day^−1^). However, in that study, the semicontinuous cultivation phase lasted less than 15 days, was conducted during a high-productivity season (spring), employed nearly twice the nitrate concentration (0.9 g L^−1^ NaNO_3_), and involved no medium recycling. In the present study, a comparable maximum areal productivity of 15.1 gDW m^−2^ day^−1^ was recorded during P5 (day 488–489; 29–30 June 2022—summer), after 60 days of continuous cultivation in full seawater. It should also be noted that, although surface area and culture depth were identical, the raceway ponds used by Villaró et al. [12] had a larger length-to-width ratio (20 vs. 3.6 in this study) and were equipped with a sump with continuous air injection, likely providing improved hydrodynamics [59] and oxygen removal [60], which may have enhanced biomass productivity.

By contrast, González-Portela et al. [24] reported substantially higher productivities (35–60 gDW m^−2^ day^−1^) during an 8-month cultivation of *L. maxima* in full seawater in Saudi Arabia, using 10,000 L open-air raceway ponds without shading, although the culture medium was reused for only 3–5 cycles, unlike the present study. These values rank among the highest reported for the genus *Limnospira* and for microalgae in general, approaching the maximum photosynthetic conversion efficiency of 5% proposed for outdoor microalgal cultures [61,62]. This may suggest a complete acclimation of the strain to the high salinity (42 g L^−1^) and high irradiance (average: 2,500 µmol m^−2^ s^−1^) conditions prevailing at that location. On the other hand, according to the results of the present study, although no significant differences in overall productivity were observed between shaded (P6) and non-shaded (P6NS) optimized full-seawater conditions for *L. platensis* BEA 1257B, the phase-specific analysis according to pigmentation state revealed lower productivity, C-PC content, and Fv/Fm during the pale-green stage under non-shaded operation, which later approached those observed in P6 following a controlled reduction in culture salinity. This discoloration was not observed under freshwater cultivation conditions [3], possibly suggesting that high light availability for photosynthetic reactions could be a limiting factor under the tested full-seawater conditions. Therefore, further work on strain acclimation to high irradiance under saline stress is warranted, although it should be considered that different *Limnospira* strains may exhibit markedly different acclimation capacities [12,15,24]. Another potential strategy to enhance the productivity of this strain would be the implementation of automated shading systems that dynamically adjust to external irradiance, providing protection only during periods of excessive light exposure [3,27,63,64].

In this study, the culture was deliberately maintained at high biomass densities throughout all experimental phases to allow progressive acclimation to salinity, discourage the growth of other organisms (see Section 3.1), and promote self-shading of the cells. In dense cultures, this condition was expected to mitigate exposure to extreme UV radiation and high ambient temperatures, thereby reducing the risk of excessive stress. Semicontinuous cultivation cycles were generally operated at biomass concentrations between 0.8 and 1.0 gDW L^−1^, in line with Kurpan et al. [63] and Guerra et al. [64], which are approximately two-fold higher than those commonly reported in the literature [12,24,27,52]. Although this strategy may have partially limited overall culture productivity, operating at higher biomass concentrations at harvesting can reduce the energy use per unit of harvested biomass by more than 50% [3], representing a significant opportunity for cost reduction, given that harvesting alone can account for ≃25% of total cultivation costs in raceway ponds due to the typically low biomass densities achieved in such systems [65].

Culture shading and the use of a full-seawater medium also represent effective strategies to reduce water evaporation. This is supported by the 34% lower evaporation rate (ER) observed in P6 compared to P6NS, likely due to the lower culture temperatures reached during periods of highest irradiance, as well as by the decreasing trend in ER observed with increasing culture salinity under shaded conditions. This effect could potentially be related to the influence of dissolved salts on water vapor pressure, as the interaction between water molecules and dissolved ions may reduce their tendency to transition into the vapor phase [66]. The daily ER measured in the optimized full-seawater conditions (P6_Total_: 2.4 ± 1.2% day^−1^, corresponding to 3.0 ± 1.5 mm day^−1^) was consistent with that reported by Guidi et al. [3] (3.0 ± 1.1 mm day^−1^) in the same raceway system and greenhouse, who observed reduced ER values compared with open ponds located in arid or semi-arid regions (average 6 mm day^−1^; [47,67,68], likely as a result of increased relative humidity in the surrounding atmosphere provided by the greenhouse structure. This reduction in water loss corresponds to an approximately 50% decrease in evaporation-related water demand, considering that evaporation typically accounts for more than 35% of the total water demand in freshwater microalgae cultivation systems [69]. In the P6_Total_ phase, water evaporation is more than three-fold lower than the values reported for open-air raceway ponds cultivating *L. maxima* in full seawater in Saudi Arabia (8% day^−1^; [24]).

When comparing the overall average productivity of *L. platensis* BEA 1257B under optimized full-seawater conditions (0.033 ± 0.014 gDW L^−1^ day^−1^, corresponding to 4.1 ± 1.4 gDW m^−2^ day^−1^; range: 0.011–0.086 gDW L^−1^ day^−1^, equivalent to 1.4–10.8 gDW m^−2^ day^−1^) with other long-term studies on *Limnospira* cultivation in freshwater in Europe, the values reported here were 20–25% lower than those obtained in a year-round greenhouse study conducted in central Portugal using large-scale raceway ponds (1000–4000 m^2^; 5.1–5.6 gDW m^−2^ day^−1^; [24]), with a comparable productivity range. Moreover, the average volumetric productivity observed in the present study was similar to the maximum value of 0.038 gDW L^−1^ day^−1^ reported by Pistocchi et al. [27] during a 3-month cultivation period under best-productivity season (May–August) in northern Italy, conducted in 60 m^2^ greenhouse raceway ponds, while the maximum areal productivity achieved here was more than 40% higher than the 7.6 gDW m^−2^ day^−1^ reported in that study. In contrast, the average areal productivity obtained here was approximately half of the 12-month average reported by Jiménez et al. [16] for *L. platensis* cultivated in Málaga in a 450 m^2^ open-air raceway pond at 0.30 m culture depth (8.2 gDW m^−2^ day^−1^), although the average volumetric productivity in the current study was about 20% higher (0.027 gDW L^−1^ day^−1^ in Jiménez et al. [16]). Similarly, the areal productivity reported here was approximately one-third of that achieved in a 12-month cultivation study conducted in northern Italy under a heated greenhouse [63]. While overheating is generally not a major concern for *Limnospira* cultivation in open raceway systems during summer, even under greenhouse conditions, temperatures below 15–17 °C can strongly limit productivity for several months of the year in temperate regions [9,16]. The three-year continuous production of *L. platensis* achieved in this study without additional heating costs highlights the Canary Islands as an attractive location for year-round cultivation, in comparison with other regions of Europe, Asia, and North America, where non-heated *Limnospira* cultivation is typically limited to 6–7 months per year [27,47,70]. This advantage is particularly relevant in the context of entering the market with fresh, refrigerated, or frozen biomass, which represents an emerging trend for new commercial *Limnospira* products [68].

### 3.3. Biomass Composition Under Optimized Full-Seawater Conditions

The biochemical composition of the spray-dried biomass obtained in P6_Total_ revealed a protein content of 46.4 ± 4.2 g 100 g^−1^, consistent with values reported for *Limnospira* cultivated under long-term full-seawater conditions, ranging between 40 and 55% [21,24], although slightly lower than the protein content of >50% typically reported for commercial *Limnospira* products [6]. This value is 25% lower than that reported for the lyophilized biomass of the same strain grown in freshwater (62.5 ± 2.9 g 100 g^−1^; [3]). However, this difference is limited to 19% when samples are compared on an AFDW basis. Taking into account that this genus has never been reported as naturally occurring in marine environments [21,52], this moderate reduction in protein content was expected as a consequence of prolonged exposure to high salinity. This latter has been shown to constrain nitrogen assimilation and promote metabolic reallocation towards osmoprotective compounds, such as carbohydrates and soluble sugars, as part of the cellular strategy to maintain osmotic balance and essential cellular functions under ionic stress [21,64]. Synergistically with salinity, prolonged medium recirculation may have contributed to the observed protein decrease, likely due to progressive nutrient imbalance, accumulation of inhibitory metabolites, and altered nitrogen availability over time [63,71,72]. This could explain the lower protein content reported here in comparison with another *L. platensis* strain cultivated in full seawater over a relatively short cultivation period (1 month) with no medium recycling (60.9 ± 2.0 g 100 g^−1^) [12]. In fact, according to ANOVA and correlation analyses, reducing nitrate supply in the make-up medium from 2.0 (P1) to 0.5 g L^−1^ of KNO_3_ (P2 and P6 onwards) did have a weak effect on the biomass protein content and may have partially contributed to this difference. This is consistent with previous findings for *Limnospira* under N-limited conditions, where carbon allocation tends to shift toward the synthesis of carbon-rich compounds as the cellular C–N ratio increases [73,74]. Notably, the higher protein levels reported by Villaró et al. [12] were achieved under a culture medium supplying approximately two-fold more nitrogen than the optimized full-seawater medium used in this study (0.15 and 0.07 g N L^−1^, Villaró et al. [12] and this study, respectively).

Consistent with the effect of prolonged salinity exposure, the 8% protein decrease observed during P4 compared to P1 (10% seawater condition) closely aligns with the ≃5% decline reported by Villaró et al. [12] after 1 month of cultivation in seawater. Homeostasis, i.e., the capacity to preserve cell composition despite external disturbances, is most likely under brief perturbations (relative to the organism’s growth rate), when reallocation of cellular resources does not confer a meaningful reproductive advantage, and avoiding subsequent reverse acclimation once the perturbation subsides is energetically preferable. Acclimation, by contrast, which requires extensive remodeling of the expressed proteome, is more likely to occur under prolonged stress [75]. Seasonal effects could also have contributed to these differences, as protein measurements in P4 were taken in winter, whereas Villaró et al. [12] conducted their trial in spring. Winter-associated reductions in protein content have been previously reported for *Limnospira* [24,63]. Nevertheless, protein content during the 615-day-long P6_Total_ phase notably showed relatively limited variability, suggesting stable levels for this cellular macrocomponent once full-seawater conditions were established. Stable protein contents for this strain were previously reported by Guidi et al. [3] in freshwater, both with fresh and recirculated medium. Therefore, the protein contents for *L. platensis* BEA 1257B seem more related to culture-specific factors, such as the physiological adaptation of the strain to salinity, different culture medium, or pH-related effects, rather than to seasonal variability [76,77], in line with year-round studies conducted in southern Spain and Northern Italy that reported relatively stable protein contents throughout the year [3,27,64].

According to the negative and positive correlations of protein and carbohydrate content, respectively, with salinity, the shift in biomass composition was also reflected by the higher carbohydrate content in the spray-dried P6_Total_ biomass (17.2 ± 7.3 g 100 g^−1^) compared with P1 and P2 phases, in line with the osmoadaptive accumulation patterns observed in other seawater-based cultures [12,24,64]. Nevertheless, this carbohydrate content is in the range of common commercially dry *Limnospira* (17–25%; [6,35]) and similar to that obtained for the same strain in freshwater with recycling of the culture medium (17.2 ± 1.6 g 100 g^−1^; [3]).

In line with the general preference of this cyanobacterium to accumulate carbohydrates over lipids as carbon storage pools [78], lipid content in the spray-dried biomass remained generally low (<5.2%), averaging values of 3.4 ± 0.6 g 100 g^−1^ in P6_Total_, which are slightly below the range of common commercially dry *Limnospira* (4–8%; [6,35]) and the levels obtained for the same *Limnospira* strain in freshwater (4.2–7.4%; [3]). According to GLM, these low levels did not appear to be associated with the salinity increase and/or solar irradiance, suggesting that other cultivation factors may have constrained lipid accumulation. A reduction in lipid content was reported by Guidi et al. [3], who observed a 40% decrease (from 7.4 ± 0.7 to 4.2 ± 0.4%) following the initiation of medium recycling. Although the authors of that study hypothesized that lipid reduction was primarily related to high pH rather than to medium recycling, the data obtained here suggest the opposite. In fact, while average pH and lipid content across phases did not display a clear relationship, lipid content showed a slightly decreasing trend from phases P1 to P6_Total_ under medium recycling, followed by the highest values in P7, when the fresh medium was supplied. Nevertheless, GLM analysis identified a positive association between the frozen-biomass processing method on lipid content, indicating that part of the observed variability may be attributable to downstream processing rather than to cultivation conditions alone. Further studies are needed to clarify the potential role of medium recycling in modulating biochemical macrocomponents of the biomass under long-term cultivation.

C-phycocyanin in this study showed a moderate negative correlation with salinity, with the highest contents observed in 10% seawater (P2: 12.4 ± 2.1% AFDW). This supports previous findings claiming that cultivation at low-to-intermediate salt concentrations (5–10 g L^−1^) favors the accumulation of both protein and phycocyanin in *L. platensis* biomass [21]. C-PC content in P6_Total_ (9.64 ± 2.69 g 100 g^−1^) was higher than that obtained for the same strain cultivated in freshwater (7.2 ± 1.1%; Guidi et al. [3]) and similar to the values reported for an Indian *L. platensis* strain grown outdoor in seawater in southern Spain (9.4 ± 0.04 g 100 g^−1^; Villaro et al. [12]) and to other strains cultivated in freshwater [16,76]. However, it was slightly lower than the 10–17% reported elsewhere [27,35,64,79]. Phycocyanin content is often reported to decrease under saline stress because of the downregulation of photosynthetic antenna complexes, a response associated with energy saving and mitigation of oxidative damage [64]. Although not statistically significant, lower C-PC contents were recorded in 10% seawater cultures under shaded conditions during winter–spring (P1) compared to spring–summer (P2), with the former phase characterized by significantly lower irradiance and culture temperatures than the latter, suggesting that seasonal factors may also exert a certain influence on phycocyanin accumulation. Similar results were also observed in full seawater, where lower C-PC contents were recorded under shaded conditions during autumn–winter (P4: 8.3 ± 1.8% AFDW). This seasonal decrease is consistent with previous studies reporting lower phycocyanin contents in biomass produced under reduced temperatures and lower solar irradiance [16,64,76]. Nevertheless, the lowest levels of C-PC were found here under non-shaded conditions year-long (P6NS: 6.7 ± 1.5% AFDW), which exhibited a 38% lower C-PC content than P6_Total_ within the same temporal framework. This finding points out that high irradiance may represent a major limiting factor for phycocyanin accumulation, especially under salinity stress, as previously described for productivity (see Section 3.2), due to pigment degradation [64,76,80,81]. Therefore, the relatively high C-PC content maintained in P6_Total_ may have been favored by the use of a shading net, which likely reduced photoinhibition and oxidative stress under full-seawater conditions [37]. The divergence between relatively stable total protein content and more variable phycocyanin levels possibly reflects a regulated cellular resource-allocation strategy under physiological stress. While overall protein content may remain stable, *Limnospira* selectively modulates specific protein fractions, reducing the synthesis and partially catabolizing energetically costly phycobilisomes to conserve energy and remobilize nitrogen under environmental stress [64].

Ash content in P6_Total_ (15.6 ± 7.6 g 100 g^−1^) was approximately 1.5-fold higher than that of freshwater *Limnospira* powders currently available on the market. Nevertheless, it remained within the range of 10–17% reported for *Limnospira* cultivated in other arid and semi-arid regions [16,82] and in full-seawater media (10–17% in [12] and 11% in [24]). Moreover, this value was about half of the ash content reported for marine species cultivated at the same site with the same cultivation systems but harvested by centrifugation [26,31]. The relatively high ash content in P6_Total_ likely depends on both medium salinity and recirculation [63]. Indeed, salts not assimilated by the cells could account for up to 20% of the ash content in the harvested dry biomass [12,63,64]. In this study, ash content in the spray-dried biomass under optimized full-seawater conditions occasionally reached values as high as 20–25%. However, this amount was significantly reduced by implementing the R_mod_ approach, ultimately achieving levels between 5 and 8%. These values are in the lower range of values reported for commercial *Limnospira* biomass (6.4–13%; [35,79,83]), indicating the effectiveness of the developed method.

The increase in salinity resulted in a general rise in mineral content of the biomass, especially of K, Na, Mg, Ca, Zn, B, and As, consistent with trends previously reported for *L. platensis* cultivated under saline conditions [12,64]. K and Na emerged as the predominant essential elements at all phases, in line with the characteristic mineral profile reported for the genus *Limnospira* [3,5,12]. K was similar in P1, P4, and P6_Total_ (10, 100, and 100% SW, respectively) while being significantly lower in P3 (50% SW). This possibly reflects the total amount of K supplied to the culture medium, which originated from the addition of both KNO_3_ and natural seawater (485 mg L^−1^ at 100% SW). Indeed, based on these inputs, the estimated K concentration in the initial make-up medium reached approximately 819 mg L^−1^ in P1 (10% SW, 2 g L^−1^ KNO_3_), decreased to 628 mg L^−1^ in P3 (50% SW, 1 g L^−1^ KNO_3_), and stabilized around 678 mg L^−1^ under optimized full-seawater conditions in P6_Total_ (100% SW, 0.5 g L^−1^ KNO_3_). The K content measured in the spray-dried biomass of *L. platensis* BEA 1257B cultivated under optimized full-seawater conditions fell within the range reported for the same strain and for other *Limnospira* strains grown in freshwater [3,35,79,84], as well as for *L. platensis* cultivated in seawater [12]. This consistency across studies highlights that cultivation in seawater does not compromise the potassium level of *Limnospira*, supporting its suitability for human nutrition in line with WHO recommendations encouraging higher dietary potassium intake [85].

On the other hand, Na content reached 2822 ± 2460 mg 100 g^−1^ DW in P6_Total_ (corresponding to 2371 ± 2518 mg 100 g^−1^ FW), four-fold higher than in 10% seawater (P1). Although NaHCO_3_ and Na_2_CO_3_ in the culture medium were progressively reduced, this reflects the net rise in Na concentration due to the NaCl contained in seawater. Actually, Na levels were expected to be around 11,348 mg L^−1^ in the 100% SW medium supplemented with 0.6 g L^−1^ of both bicarbonate and carbonate, which is nearly three times those of P1 (4452 mg L^−1^; 10% SW and 8 g L^−1^ NaHCO_3_) and twice those of P3 (6316 mg L^−1^; 50% SW and 1 g L^−1^ of each carbonate salt). The Na concentration in the P6_Total_ spray-dried biomass was 47% higher than that of the same strain cultivated in freshwater [3] and almost twice that of values previously reported elsewhere for *Limnospira* cultivated in freshwater [35,79,84]. Still, Na content here was ≃40% lower than another *L. platensis* strain grown in full seawater [12] and similar to that of a marine species cultivated at the same site with the same cultivation systems [26]. Overall, Na levels remained within the upper range of commercial *Limnospira* products and within acceptable ranges for dietary applications [86]. The high variability observed in Na content in P6_Total_ is likely attributable to the initial ineffective biomass washing, which resulted in the inclusion of samples with different residual salt content. This also indicates that Na levels can be significantly reduced through improved and standardized washing procedures. In particular, implementing the R_mod_ step at each harvest batch would enhance the removal of residual salts from the algal slurry (see also above) and improve the reproducibility of the final powder composition. Such post-harvest strategies represent a practical approach to mitigate Na accumulation in biomass produced under seawater-based cultivation conditions, thereby better aligning with recommendations to reduce sodium intake from food [86]. In fact, preliminary results from recent production batches (2025–2026) obtained under R_mod_ step showed substantially lower sodium contents in the spray-dried powder (1500–1700 mg 100 g^−1^ FW; mean 1433 ± 379 mg 100 g^−1^ FW) and ash levels (6.81–7.50 g 100 g^−1^ FW; mean 6.87 ± 0.60 g 100 g^−1^ FW), supporting the effectiveness of these optimization strategies.

Ca and Mg followed the same trend described for Na, both being four-fold higher in P4 and P6_Total_ than in P1, because of the progressive enrichment of seawater in the culture medium. Throughout the experiment, Ca and Mg were neither supplemented in the make-up medium nor replenished during medium recycling, so seawater represented the primary source of these ions (approximately 1320 mg L^−1^ Mg and 445 mg L^−1^ Ca in full seawater), followed to a much lower extent by desalinated water to compensate for evaporation losses (see Section 3.1). Interestingly, the highest, although not significantly different, values in the biomass were observed in P3 (50% SW), possibly as a result of precipitation of these ions occurring at culture pH values above 9.5 (see also Section 3.1). Ca content in P6_Total_ spray-dried biomass fell within the range documented for *Limnospira* in freshwater [35,79,87]. Conversely, it was five-fold lower than the value reported for a seawater-grown strain [12]. In contrast, Mg content was between two- and four-fold higher than values previously reported for *Limnospira* cultivated in either freshwater [3,35,79,84,87] or seawater [12], making *L. platensis* BEA 1257B cultivated in full seawater a promising source of this mineral for food and nutraceutical applications.

Fe was the most abundant trace element across all phases, consistent with the general profile of *Limnospira* biomass and microalgae in general. Interestingly, Fe content in the biomass decreased proportionally with increasing salinity, with P6_Total_ containing approximately half the content of P1 and the same strain cultivated in freshwater [3]. As a result, Fe content in P6_Total_ (32.4 ± 18.1 mg 100 g^−1^ DW) is roughly half of the previously reported values in *Limnospira* biomass [3,35,79,84]. A significantly lower Fe content has previously been documented in full-seawater cultures of *L. platensis* when compared to freshwater cultures [12], likely due to partial precipitation under high-salinity conditions driven by elevated Ca and Mg concentrations [3]. Moreover, lower Fe contents have likewise been reported under medium recirculation, suggesting that Fe may be consumed faster than it is replenished [26]. This may explain the lower Fe content observed here under optimized full-seawater conditions compared with that reported by Villaro et al. [12], in which medium recirculation was not applied. Fe limitation may have indirectly contributed to the lower protein content observed by constraining photosynthetic performance and nitrate assimilation [88]. Further optimization by increasing Fe concentration in the culture medium is warranted. Nevertheless, the Fe content in P6_Total_ underscores its suitability for addressing iron-deficiency anemia, particularly in populations with limited access to dietary iron, a situation commonly observed in resource-limited regions of arid and semi-arid coastal areas characterized by restricted dietary diversity [89,90].

Mn, Zn, and B exhibited significantly higher concentrations in the biomass under optimized full-seawater conditions compared to P1, with four- to six-fold increases. Values measured in P6_Total_ exceeded the upper ranges previously documented for the genus *Limnospira* [3,35,79,84]. From a nutritional perspective, 100 g of spray-dried P6_Total_ biomass would be sufficient to provide more than twice the daily Nutrient Reference Value (NRV) for Mg and Mn, approximately 100% of the NRV for Fe, and over 15% of the NRV for Ca. Although consumption of 100 g of *Limnospira* biomass per day is unrealistic, there is no legally mandated maximum daily intake established at the European Union level, and daily intakes in the range of 3–10 g are commonly reported in clinical studies and commercial recommendations, with higher intakes occasionally explored in specific contexts [91,92]. Altogether, these results support the strong potential of this biomass for incorporation into food products aimed at achieving the 15% NRV threshold required for nutrition claims under EU legislation [93].

Although their concentrations tended to increase with salinity, low heavy metal levels were detected under all tested conditions. Pb, As, Cd, and Hg were quite below the limits set by the EU regulations for undesirable substances in food and feed applications, and also for heavy metals in food supplements [94,95,96]. When comparing *L. platensis* BEA 1257B under optimized full-seawater conditions with that cultivated in freshwater at the same site and using the same cultivation system [3], significantly higher As content was found in the former condition. This is likely associated with the higher background concentration of this metal in seawater and its accumulation during prolonged medium recirculation combined with evaporation compensation [26]. Notwithstanding, As content in P6_Total_ (0.23 ± 0.17 mg kg^−1^ DW) was well below levels reported for edible seaweeds (1–100 mg kg^−1^ FW) and for spinach and other leafy vegetables (0.5–2 mg kg^−1^ FW; [97,98,99]), and comparable to marine microalgae cultivated in the same site and cultivation systems with long-term medium recirculation [26].

High relative abundances of the essential fatty acids LA and GLA (>20% of total FA each) were detected under the optimized full-seawater conditions (P6_Total_). This observation is consistent with the recognized tendency of *Limnospira* to increase unsaturated C18 fatty acids under ionic stress, possibly as an adaptive response to maintain membrane fluidity [64]. Absolute abundances of LA and GLA were comparable to those reported for another *L. platensis* strain cultivated in full seawater in southern Spain [12], which showed a reduction relative to freshwater conditions. Nevertheless, the fatty acid profile of *Limnospira* is known to be highly strain dependent; for example, linoleic and γ-linolenic acid contents were reported to range between 13–32% and 13–29% of total fatty acids, respectively, across 35 different *Limnospira* strains [100]. As expected for cyanobacteria, long-chain PUFAs such as EPA and DHA remained below detection limits (<0.05%; [35,64]).

The amino acid profile obtained under optimized full-seawater conditions was broadly consistent with those commonly reported for commercial *Limnospira* [6,35] and comparable to that of other seawater-based studies [12,21]. The relative abundance of essential amino acids exceeded the FAO adult protein nutritional score patterns [101] for leucine, valine, isoleucine, threonine, and tryptophan, while lysine and histidine were slightly below the threshold. The authors detected only minor changes in the amino acid profile when comparing freshwater- and seawater-grown biomasses, indicating that although long-term seawater cultivation slightly affected protein content, it did not compromise the essential amino acid profile and protein quality, confirming *L. platensis* cultivated in full seawater as a suitable alternative protein source in arid and semi-arid coastal areas.

High microbiological quality was maintained throughout the entire experiment. Total aerobic mesophilic flora remained low (10^2^–10^4^ cfu g^−1^), with no apparent trends associated with long-term seawater cultivation or medium recirculation. Yeasts and molds, as well as the screened bacterial pathogens (Enterobacteriaceae, total coliforms, *Escherichia coli*, *Staphylococcus* spp., *Clostridium perfringens,* and *Salmonella* spp.), were generally not detected or below the detection limits of the analytical methods. Overall, the microbiological characteristics of the spray-dried *L. platensis* BEA 1257B biomass cultivated under optimized full-seawater conditions comply with current food safety criteria for microalgae intended for human consumption [3,79,102]. Although microbiological analyses were conducted in accordance with current food safety standards for microalgae biomass, further investigation of marine-specific microorganisms (e.g., *Vibrio* spp.) and viral agents such as bacteriophages could provide additional insights into culture stability and long-term process robustness in seawater-based cultures. However, it is well established that the highly alkaline conditions maintained during *Limnospira* cultivation (pH > 9) limit the proliferation of many contaminating and potentially pathogenic microorganisms [9]. Furthermore, recent studies have shown that increases in pH and dissolved oxygen in marine systems can significantly reduce the abundance of *Vibrio* spp. [103]. Additionally, downstream processing steps such as spray-drying may contribute to further microbial load reductions in the final product. Moreover, the biomass tested negative for PAHs, pesticides, and mycotoxins, confirming its safety according to EU food safety standards for *Limnospira* as a dietary supplement [6,104]. Furthermore, no crustacean, fish, or mollusk DNA was detected, indicating that the biomass is free from potential allergen contamination associated with seawater cultivation. To the best of our knowledge, this is the first study reporting this type of food safety analysis in the dried biomass of *L. platensis* cultivated in full seawater.

### 3.4. Environmental and Economic Perspectives of Cultivation in Seawater with Long-Term Medium Recirculation

In the Canary Islands, the availability of freshwater is constrained by desalination capacity. Reverse-osmosis systems require chemical cleaning and generate concentrated brine as a residual stream, exerting pressure on marine ecosystems [26,105]. Moreover, the average energy demand for desalinated freshwater production has been estimated at 3.91 kWh m^−3^ [106], whereas in the present study, only 0.25 kWh m^−3^ was required to pump seawater from a borehole for *L. platensis* cultivation. The SWR strategy reduced the total energy requirement for the whole biomass cultivation process by 10.5% compared with FWR on a surface basis, mainly by eliminating freshwater use for make-up medium preparation, which accounted for a 12% decrease in the total freshwater consumption. However, when results were normalized per unit of product, these advantages were partially offset by the lower biomass productivity achieved under SWR conditions, highlighting a trade-off between productivity and resource efficiency (see Section 2.4). In particular, this effect was most pronounced when normalization was based on protein production, due to both reduced biomass productivity and lower protein content, whereas normalization based on phycocyanin production yielded consistently positive savings. At the same time, these savings remain constrained by evaporative losses, emphasizing the need to explore strategies to reduce evaporation or alternative approaches, including compensating culture volume with seawater within the salinity tolerance of *L. platensis*.

When considering fertilizer inputs for the preparation of the make-up medium, reductions of 14.0 t year^−1^ for KNO_3_ and 0.12 t year^−1^ for NH_4_H_2_PO_4_ were estimated under SWR for a 10 ha facility. While part of these savings is reduced when expressed per unit of protein produced, a consistent decrease in fertilizer demand remains, particularly for carbonate salts and NaCl, which were reduced by up to 67.7% and 100%, respectively. When expressed per unit of biomass and phycocyanin produced, these reductions further contributed to a substantial decrease in nitrate-related production costs, as nitrate prices have been steadily increasing, thereby driving the exploration and adoption of lower-cost alternative nitrogen sources in *Limnospira* cultivation [24,107]. Consequently, although SWR does not drastically reduce freshwater demand, it significantly reduces fertilizer inputs, enhancing both economic and environmental sustainability, while also decreasing reliance on imported fertilizer, in line with EU strategies [26,108]. It should be noted that the FWR benchmark values of biomass productivity, protein, and phycocyanin content used for product-based comparison represent favorable operating conditions, as they were obtained over a relatively short cultivation period (1 month of medium recirculation) and under higher nutrient supply (fourfold and two-fold higher nitrate and phosphate concentrations, respectively), which would not allow effluent compliance with current discharge regulations, thus limiting the practical applicability of this reference scenario. In contrast, the SWR strategy evaluated in this study was operated over 615 days under reduced nutrient input while maintaining effluent quality within regulatory limits. In addition, the protein content of SWR biomass still shows potential for improvement, as ash content could be reduced through the implementation of the R_mod_ washing strategy, thereby increasing the relative protein fraction.

Beyond resource savings, the most relevant advantage of SWR lies in the substantial reduction in nutrient and ion discharge to the environment. On a surface-based basis (10 ha), the implementation of this approach prevented the discharge of 1.94 t of nitrate, 0.2 t of phosphate, and 36.4 t of chloride annually, which is particularly relevant in regions with strict environmental regulations and limited options for effluent management. Importantly, these reductions remained consistently high when normalized per unit of biomass, protein, and phycocyanin produced (79.3–96.5% for N and 72.5–86.1% for P), indicating that the environmental benefits of SWR are largely independent of productivity differences between cultivation strategies.

While nitrogen, phosphorus, and chloride fluxes are commonly assessed when evaluating nutrient discharges, the impact of other major ions in the conventional *Limnospira* culture medium, such as sodium (Na^+^) and carbonates (CO_3_), is often overlooked. Similarly to Cl^−^, Na^+^ is negligibly assimilated by *L. platensis*. Moreover, under the open cultivation systems adopted in this study, inorganic carbon consumption in the culture medium due to bicarbonate uptake and assimilation is expected to be largely compensated by atmospheric CO_2_ exchange, as supported by the stable culture pH in the absence of external CO_2_ supply and the lack of productivity enhancement when CO_2_ was supplied during phase P7.CO_2_^+^. Consequently, Na and carbonate concentrations at discharge can be assumed to remain comparable to initial levels. In FWR, Na^+^ and Cl^−^ are externally added in the form of NaCl and subsequently discharged, whereas in SWR, they originate from seawater itself and return to the marine environment. Although some external carbonate supplementation is still performed in SWR, its contribution is markedly lower than in FWR since part of the alkalinity demand is supplied by the naturally dissolved carbonate/bicarbonate in seawater. Under these assumptions, SWR would prevent the annual discharge of approximately 41.4 t of Na (83.0%) and 23.2 t of carbonates (67.7%). Additionally, it should be noted that these salts significantly increase the conductivity and alkalinity of the effluent in FWR. This characteristic has important regulatory implications, making it unsuitable for direct discharge into conventional wastewater treatment systems or for use in irrigation without prior extensive dilution with freshwater (see also Section 3.1). In fact, while SWR showed a mean pH of 9.35 ± 0.4, complying with the effluent discharge limit in the Canary Islands (9.5; [43]), reported pH values for FWR (>10 on average, [3,27,35]) exceeded legal limits and therefore require neutralization prior to discharge, typically through CO_2_ injection or acid addition, increasing operational costs and environmental burden [47].

Most studies on freshwater-based *Limnospira* cultivation systems do not explicitly consider effluent management [3,27,63,64]. Considering the combined environmental, operational, and regulatory implications, SWR emerges as a robust and promising strategy for long-term sustainable and regulatory-compliant *Limnospira* production in coastal regions. A comprehensive life cycle assessment (LCA) and life cycle costing (LCC) of the SWR process is beyond the scope of this study but is warranted to fully evaluate its viability compared with FWR. Ongoing work is addressing these aspects using the data generated under realistic operating conditions.

Taken together, these findings indicate that the SWR cultivation strategy is particularly suited for regions with scarce freshwater requiring desalination. In such contexts, seawater-based *Limnospira* cultivation systems should integrate seawater intake and effluent return to the marine environment. Future development could involve coupling cultivation facilities with desalination plants, enabling the use of existing intake and outfall infrastructures, desalinated water to compensate evaporative losses, and co-discharge of culture effluents with desalination brine, potentially reducing its salinity and environmental impact. This integrated approach, already technically feasible in the Canary Islands, could be readily extended to other arid and semi-arid coastal regions, supporting sustainable marine-based food production under freshwater scarcity.

## 4. Materials and Methods

### 4.1. Strain Collection and Cultivation Maintenance

*Limnospira platensis* BEA 1257B was isolated by the Spanish Bank of Algae (BEA, Telde, Gran Canaria, Spain) from water samples taken from the Los Molinos reservoir of the Betancuria Rural Park in Fuerteventura Island (Spain, 28°30′26″ N, 14°01′47″ W) in June 2014. The reservoir contains shallow, brackish water and serves as a resting spot for a wide variety of migratory birds, which could have historically introduced the cyanobacterium from Africa [109]. The species identity was confirmed through morphological analysis using optical microscopy and by sequencing the 16S rRNA gene (GenBank MT426015). The strain naturally exhibits both linear and spiral trichome forms and was initially cultured in *Limnospira* medium [110]. At the Canarian Institute of Technology (ITC), the strain was transferred and gradually acclimated from its enriched maintenance medium to a simplified and cost-effective freshwater-based medium developed in-house, known as the OUT medium (Table 11; [3]). In the present study, the strain was acclimated indoors to a medium containing 10% *v/v* natural seawater named OUTs 10% (Table 11) and scaled-up aseptically in a growth chamber at a temperature of 25 ± 1 °C, under continuous cool white light at a photosynthetic photon flux density of 300 µmol photons m^−2^ s^−1^, and with agitation provided by bubbling air supplemented with 1% CO_2_. Natural seawater used for medium preparation was pumped from a borehole at the ITC facilities.

### 4.2. Outdoor Cultivation and Seawater Acclimation

#### 4.2.1. Cultivation Site Characteristics

The experiments were conducted from 25 February 2021 to 15 October 2024 at the ITC facilities located in Pozo Izquierdo, southeast of Gran Canaria, Spain (27°48′52″ N, 15°25′25″ W). This semi-arid coastal region experiences a subtropical climate, with high solar irradiance, over 10 h of daylight, warm temperatures (mean daily temperatures ranging from 18–25 °C), and minimal rainfall (annual average <100 mm) throughout the year. Prevailing winds, primarily from the NNE, are stronger in the summer [3]. Occasionally, SE winds bring elevated dust levels (Calima) into the area. Outdoor cultivation was carried out in RWs situated in a 1500 m^2^ greenhouse. The greenhouse structure is made of high-transparency corrugated polycarbonate (Suntuf^®^ Plus, Palram Industries Ltd., Ramat Yohanan, Israel), and overheating (>35 °C) is mitigated using fan extractors. It should be considered that solar radiation inside the greenhouse was, on average, 28% lower than under open-air conditions, according to direct light measurements recorded simultaneously using a Hansatech Quantitherm Lightmeter (Hansatech Instruments, Norfolk, UK) outside the greenhouse and at seven different locations inside the facility during August and September 2019 [31].

#### 4.2.2. *L. platensis* BEA 1257B Scale-Up Process

The *L. platensis* BEA 1257B indoor inoculum in the OUTs 10% medium was scaled-up outdoors to a maximum culture volume of 10,000 L, maintaining a dilution ratio no greater than 1:5 at each successive volume increase. This protocol averts light stress on the culture and minimizes biological contamination [35,49]. The scaling up process was conducted following the method of Guidi et al. [3]; briefly, 20 L of inoculum from the indoor cultivation facility was transferred to a 100 L bubble-mixed column photobioreactor with a 50% reduction in incident light intensity by shading until the culture reached an optical density of 0.2 at 750 nm (OD_750_; HACH Lange DR3900 UV/visible spectrophotometer; Hach Company, Loveland, CO, USA). Once the culture reached an OD_750_ of 0.8, it was fully transferred into two 250 L RWs. Then, a culture volume of 400 L was moved to a 1600 L RW, and finally, the entire volume was used to inoculate the final RW with a surface area of 80 m^2^ (culture depth = 0.125 m, culture volume = 10,000 L). Culture mixing was provided by an eight-blade paddlewheel (Ø 1.4 m), producing an average superficial fluid velocity of 0.46 m s^−1^ at 23 rpm [3]. The maximum quantum yield of photosystem II (Fv/Fm, λ_ex_ = 630 nm) was determined by chlorophyll fluorescence measurements performed with an AquaPen fluorimeter (Photon Systems Instruments, Brno, Czech Republic). Dissolved nitrate and phosphate concentrations in the culture medium were measured before and after each harvesting event using QUANTOFIX^®^ test strips (Macherey-Nagel, Düren, Germany) [26]; gradation: 0, 10, 25, 50, 100, 250, and 500 mg L^−1^ for NO_3_^−^; 0, 3, 10, 25, 50, and 100 mg L^−1^ for PO_4_^3−^; instrumental measuring range: 10–500 mg L^−1^ for NO_3_^−^ and 3–100 mg L^−1^ for PO_4_^3−^. For nitrate determination, samples were diluted at ratios of 1:12, 1:6, and 1:3 in phases supplemented with 2, 1, and 0.5 g L^−1^ KNO_3_, respectively; dilution was performed before and after harvesting in the 2 and 1 g L^−1^ phases, and only before harvesting in the 0.5 g L^−1^ phase. All cultures were supplied with natural seawater pumped from a borehole, filtered through 1 µm, and UV-treated prior to use, either used alone (full seawater) or mixed with freshwater at the percentages established for each experimental phase (see Section 4.2.3). The average physicochemical characteristics of the seawater were as follows: conductivity (20 °C), 50,283 µS cm^−1^; pH, 8.1; total dissolved solids, 37,263 mg L^−1^; suspended solids, <1 mg L^−1^; alkalinity, 104.5 mg L^−1^ as CaCO_3_. Major ion concentrations (mg L^−1^) were: Na^+^, 10,924 mg L^−1^; Cl^−^, 21,654 mg L^−1^; SO_4_^2−^, 3026 mg L^−1^; Mg^2+^, 1320 mg L^−1^; K^+^, 485 mg L^−1^; Ca^2+^, 445 mg L^−1^; HCO_3_-, 135 mg L^−1^; Br^−^, 75 mg L^−1^. At the time of inoculation, *L. platensis* cells displayed a wavy trichome morphology [3] with an average length of 277.1 ± 93.6 µm and a width of 10.9 ± 1.1 µm (*n* = 43). No significant morphological changes were observed by microscopic observation during the experiment (Leica DMi1 microscope, 40× magnification; Leica Microsystems, Wetzlar, Germany).

#### 4.2.3. Experimental Steps Toward Full-Seawater Culture Acclimation and Optimization

Long-term outdoor cultivation was conducted in semi-continuous mode, with the medium recirculated after each harvest, aiming to maintain an optimal culture concentration range of at least 0.6–0.9 gDW L^−1^ [3]. Also, to reduce light stress, culture concentration was generally maintained in the range of 0.9–1.2 gDW L^−1^ mainly during the high-salinity phases of cultivation. However, occasional disruptions such as power outages and culture discoloration, and the repeated culture refresh operation implemented as a mitigation strategy, led to lower concentration at some points. Finally, harvesting was carried out according to staff working hours, which occasionally resulted in culture concentrations above the established range. Approximately every 3 months, the culture was transferred to a contiguous RW with identical structural and hydrodynamic properties to allow routine cleaning of the original RW from biofilm or sediment particles.

**Figure A3:**
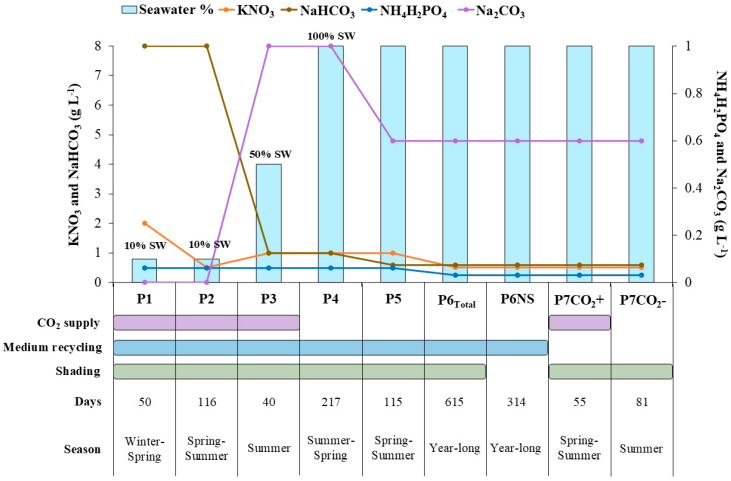
Graphical timeline summary of the experimental conditions tested for the optimization of *L. platensis* BEA 1257B semi-continuous cultures acclimated to seawater and operated outdoors under a greenhouse in 10,000 L RW, including the corresponding parameters changes as CO_2_ supply, recycling, shading, duration and seasonal period. P1 phase: OUTs10% Medium (10% *v/v* seawater); P2 phase: OUTs10% (10% *v/v* seawater) with ¼ nitrogen with respect to P1; P3 phase: OUTs50% (50% *v/v* seawater) with ½ nitrogen, ⅛ bicarbonate and 1 g L^−1^ carbonate added compared to P1; P4 phase: OUTs100% (full seawater, nutrients like P3); P5 phase: OUTs100% (full seawater, subdivided into P6.1, P6.2 and P6.3), 40% reduced bicarbonates and carbonates compared to P4; P6_Total_ phase: OUTs 100% (full seawater) with ½ nitrates and phosphates compared to P5; P6NS phase: OUTs 100% (full seawater, nutrient like P6), without shading; P7.CO_2_^+^ and P7.CO_2_^−^ phases (full seawater, nutrient like P6, no medium recirculation, CO_2_ supply and its absence, respectively).

The experiment consisted of eight cultivation phases (see medium composition and period details in Table 12 and Appendix A.4, Figure A3). An overhead shading screen was used to reduce sunlight hitting the culture surface by approximately a 53% in all phases except for P6NS, in order to reduce light stress and promote consistent growth and high phycocyanin content.

In the first phase (P1), from 25 February to 16 April 2021 (a total of 50 days), the strain performances were tested in the OUTs 10% medium.

In the second phase (P2), 16 April to 10 August 2021 (116 days), nitrogen content was reduced to one-quarter of its original concentration in the OUTs 10% culture medium (from 2 to 0.5 g L^−1^), to test the effect of a reduced nitrogen supply on biomass productivity and composition.

In the third phase (P3), seawater content in the culture medium was increased to 50% *v/v*. To achieve this, 5000 L of culture from P2 was harvested on a vibrating filter, and the resulting biomass was inoculated into a 10,000 L RW containing OUTs 50% medium. Nitrogen content was increased to 1 g L^−1^ to ensure its availability in the culture medium in case of possible precipitation that can occur at high salinity. Bicarbonate was reduced from 8 g L^−1^ to 1 g L^−1^, and 1 g L^−1^ of carbonate was added. This culture was maintained for 41 days (10 August to 20 September 2021).

Subsequently, the fourth phase (P4) transitioned the culture medium from 50 to 100% *v/v* seawater, with daily evaporation initially compensated by seawater to reach the salinity of Canarian coastal waters (37 g L^−1^), while maintaining high culture density (0.8–1.0 g L^−1^) and with the same nutrient composition as P3. This process of increasing salt concentration on a dense, physiologically mature culture (i.e., a culture already approaching stationary phase), tested here for the first time, took 16 days (20 September to 6 October 2021). Biomass productivity and composition were then assessed for 201 days in the full-seawater medium (6 October 2021 to 24 April 2022). After this period, the culture developed a milky green appearance, concomitant with precipitate formation (Appendix A.2 Figure A2).

To mitigate this, the culture was partially refreshed with OUTs 100% culture medium, where NaHCO_3_ and Na_2_CO_3_ supply was reduced by a 40% (from 1 to 0.6 g L^−1^) to minimize salt precipitation, initiating the fifth phase (P5) (115 days, 25 April to 18 August 2022). When precipitate formation was observed, in order to minimize the incorporation of mineral precipitates into the harvested biomass, paddlewheels were temporarily stopped during harvesting operations, allowing solid particles to settle before culture pumping from the surface layer.

Next, in the sixth phase (P6.1), nitrogen and phosphate supplies were reduced by half (from 1 to 0.5 g L^−1^ and 0.06 to 0.03 g L^−1^, respectively) to lower production costs and residual concentration of these nutrients in the culture effluent. These experimental conditions were tested three times: P6.1 (274 days, 18 August 2022 to 19 May 2023), P6.2 (74 days, 2 October to 5 December 2023), and P6.3 (267 days, 22 January to 15 October 2024). These phases are collectively referred to as P6_Total_ (615 days). During P6.1, 5000 L of full-seawater acclimated culture were used to inoculate an identical 10,000 L adjacent RW to test long-term cultivation without a shading screen (P6NS; 314 days, 7 September 2022 to 18 July 2023).

During the seventh phase (P7; 136 days, 19 May to 2 October 2023), productivity was evaluated under non-recirculating culture medium conditions, also assessing the effect of CO_2_ supplementation (P7.CO_2_^+^; 55 days, 5 May to 13 July 2023) versus its absence (P7.CO_2_^−^; 71 days, 13 July to 22 September 2023) with the same nutrient composition as P6. Following P7.CO_2_^−^, culture conditions reverted to those in P6 (beginning P6.2; 74 days, 22 September to 5 December 2023).

During the eighth phase (P8; 48 days, 5 December 2023 to 22 January 2024), experimental activity was temporarily suspended due to renovation work being carried out in the greenhouse near the RWs, and the culture was maintained in three 250 L RWs; data from this phase were not included in the statistical analyses. Afterward, P6 conditions were restored (P6.3; 267 days, 22 January to 15 October 2024), following the same scale-up process as at the study’s onset.

Overall, this long-term outdoor shade culture reaches a total of 1328 days in semi-continuous mode, of which 1105 days were conducted using a full-seawater culture medium.

Average daily solar radiation (G_0_, W m^−2^ day^−1^) data for this period was obtained from the Grafcan platform [111]. Specifically, meteorological records from the Santa Lucía de Tirajana, Vecindario weather station (94 m above sea level), located approximately 5 km from the ITC in Pozo Izquierdo, were selected. Culture parameters, including temperature, pH, salinity, OD_750_, and microscopic observations, were monitored on working days at approximately 9:00 a.m. and after each harvesting. Culture samples for measurements were taken in front of the paddlewheel and placed in 50 mL sterile Falcon tubes for transport to the laboratory. The biomass concentration of *L. platensis* in the culture (Cx) was determined with a regression equation to describe the relationship between dry weight (DW) measurements in g L^−1^ and OD_750_, as described by [3]. During the P1 and P2 phases, when the culture medium had 10% of salinity, the correlation equation was Cx = 0.7 × OD + 0.01, *r*^2^ = 0.993. For phases P3 and P4 and subsequent phases, when the culture medium reached a salinity of 50% and 100%, correlation equations were Cx = 0.9 × OD + 0.02, *r*^2^ = 0.993 and Cx = 1.0209 × OD + 0.01, *r*^2^ = 0.999, respectively. The correlation equations were established at the beginning of each salinity phase based on measurements obtained over at least three semi-continuous cultivation cycles.

The volumetric biomass productivity on a dry weight basis (*P_vol_*) was calculated based on the following equation:

(1) $$P_{vol}\;\left(g{DW\;L}^{-1}\;{day}^{-1}\right)=\frac{\left(Cx\;End-\;Cx\;Start\right)}{\left(t_{f}\;-\;t_{0}\right)}$$

where *Cx End* and *Cx Start* (gDW L^−1^) correspond to biomass concentrations measured immediately before and immediately after each harvest event in semi-continuous operation, respectively. Therefore, P_vol_ represents the rate of biomass concentration increase within the reactor between harvests and does not include biomass removed during harvesting (i.e., it reflects concentration-based productivity rather than total biomass production). This approach is commonly adopted in semi-continuous microalgae cultivation systems in which biomass is periodically removed to maintain a target culture density [112]. Harvested biomass was monitored during routine operation; however, gravimetric measurements in the real operational environment were not obtained with sufficient accuracy to support rigorous mass-based productivity calculations. Therefore, productivity was consistently evaluated based on concentration changes within the reactor. All culture densities and volumetric and areal productivities throughout the text are expressed on a dry weight (DW) basis.

Pure CO_2_ injection through a porous ceramic diffuser during daylight hours (12 h day^−1^) was applied during phases P1, P2, and P7.CO_2_^+^. Water evaporation in the culture was compensated daily according to measured increases in salinity using 1 µm of UV-filtered desalinated water (average Ca, Na, and Mg content: 12, 90, and 19 mg L^−1^, respectively), which was also used for medium preparation prior to the use of full seawater. All chemicals used in make-up medium preparation and nutrient replenishment were of commercial grade, sourced from local providers and selected for their low metal content and high solubility.

### 4.3. Biomass Harvesting and Processing Optimization

Biomass harvesting was performed on culture volumes ranging from 2000 to 3500 L, representing 20–35% of the total culture volume. Biomass was separated using an industrial circular vibrating screen (Filtra^®^ FTI-0800, Ø 800 mm, filtration area 0.5 m^2^; Filtra Vibración, Barcelona, Spain) equipped with a 15 µm nylon mesh mounted over a stainless-steel support mesh. The culture was pumped to the separator from the surface of the RW at a constant flow rate of 750 L h^−1^ using a peristaltic pump. Harvesting efficiency, defined as the percentage of biomass retained by the filter during harvesting, was determined based on the difference between the *L. platensis* concentration in the filtrate and in the culture [3], and averaged 89.1 ± 5.1%. The liquid filtrate was directly returned to the RW during the harvesting process in all cultivation phases except when culture was transferred to a contiguous RW for routine cleaning and for both P7 phases, where the harvested volume was replaced with an equivalent volume of fresh medium. The exhaust medium was discarded as effluent through a filtering well. Nutrients (N, P, and micronutrients, including iron) were replenished on each cycle to the initial concentration after measuring the residual concentration of N and P in the culture medium. Micronutrients were reintegrated proportionally to nitrate supplementation in order to maintain the relative composition of the original culture medium during semicontinuous operation and medium recycling (Table 12). This approach represents a practical operational strategy commonly applied in large-scale *Limnospira* and other microalgae cultivation, where micronutrient demand is generally associated with biomass production and macronutrient consumption (e.g., [26,35,113,114]). However, since micronutrient uptake is not necessarily strictly proportional to nitrate assimilation, this approach should be regarded as an operational approximation rather than a stoichiometric relationship, particularly under varying salinity and recycling conditions [113,114].

To reduce the ash content in the biomass due to the presence of residual salts from the culture medium, different post-harvesting steps of washing and dewatering were evaluated on four harvest batches of fresh biomass at the beginning of phase P1. Briefly, the harvested algal slurry was (i) neither rinsed, nor pressed (NRNP; control condition); (ii) rinsed with freshwater (5:1 *v/v* FW, corresponding to approximately 50 L of freshwater per kg of dry biomass [3]) directly onto the vibrating filter (R); (iii) manually pressed through a 30 µm nylon net (P); (iv) firstly rinsed with freshwater (5:1 *v/v* FW [3]) directly onto the vibrating filter and then manually pressed through a 30 µm nylon net (RP). During the rinsing step, batches of approximately 5 kg of fresh biomass were processed. Freshwater was applied for approximately 2 min by continuous pouring through a hose positioned on one side of the vibrating filter while the filter was operating. After rinsing, the vibrating filter was switched off, and the biomass slurry was removed manually. Step R was successively applied in all phases. However, while in phases P1 to P7 the vibrating filter was switched off immediately after completion of the rinsing step, during phase P6.3, the filter was kept operating for an additional 1.5 min after rinsing to allow further drainage of the slurry before stopping vibration and removing the biomass (R_mod_, see Results Section 2.3.1). The freshwater used for biomass rinsing was not discharged but directly returned to the RW and used to compensate evaporative losses. Therefore, rinsing water did not represent an additional freshwater input but formed part of the total freshwater volume required for evaporation compensation.

To compare the effects of different drying methods on the biochemical composition of *L. platensis*, the harvested biomass was dehydrated by either spray-drying or freeze-drying after being frozen and stored at −20 °C. Spray-dried biomass was dehydrated at a feed rate of 13 kg h^−1^ with a rotary atomizer spray dryer (Model L-12, Ohkawara Kakohki Co., Ltd., Yokohama, Japan), using an inlet air temperature of 190 °C and an outlet temperature of 85 °C. For freeze-dried biomass, the fresh algal slurry was frozen in stainless-steel trays (biomass layer thickness = 0.02 m) and processed using a Lyobeta^®^ 6PL lyophilizer (Telstar, Barcelona, Spain). The freeze-drying protocol included freezing at −40 °C for 4 h, primary drying in two steps (15 °C for 20 h, followed by 20 °C for 20 h at 200 µbar), and a final secondary drying step at 30 °C for 5 h at 800 µbar. All biomass samples were packed in food-grade polyethylene bags. Biomass obtained by both dehydration methods was stored at 25 ± 3 °C under dark conditions and in a dry environment until further analysis.

### 4.4. Biochemical and Microbiological Analysis of the Biomass

Proximate composition of the biomass was determined following established methodologies (AOAC, 2000; [115]). Protein content (calculated as N × 6.25) was determined using the Kjeldahl method. Although lower conversion factors (e.g., 4.78 and 5.95) have been proposed for marine microalgae and cyanobacteria [116], the nitrogen-to-protein conversion factor used in this study was selected to enable direct comparison of the *L. platensis* BEA 1257B protein content with values reported in the *Limnospira* literature, where it remains the standard for commercial *Limnospira* products and most published studies [3,6,26,64,116], thereby minimizing apparent differences in protein content that may arise solely from methodological variation. Crude lipid content was determined by acid hydrolysis followed by solvent extraction (gravimetric method). Moisture content was measured by drying samples in an oven at 105 °C to a constant weight, and ash content was determined by combustion in a muffle furnace at 550 °C for 12 h. Total carbohydrate content was estimated by subtracting the sum of protein, lipid, ash, and moisture percentages from the total biomass [117]. Energy content was calculated as the sum of energy contributions from crude protein, total carbohydrates, and crude lipids using their respective conversion factors of 4, 4, and 9 kcal g^−1^ [84]. To allow for a robust comparison of the biochemical composition between samples and phases, analytic data were first normalized by removing the contributions of moisture and ash, which can vary significantly depending on biomass washing step and drying method and according to the following equation:

(2) $$Normalized\;Component\;\left(\%\right)=\frac{MC\;\left(\%\right)\;\times \;100}{100\;-\;(M\;\left(\%\right)\;+\;A\;\left(\%\right))}$$

where *MC* = Measured Component percentage value of the analysis, *M* = Moisture percentage of the biomass, and *A* = Ash percentage of the total biomass.

Mineral composition was analyzed using inductively coupled plasma optical emission spectrometry (ICP-OES AVIO 500, Perkin Elmer Inc., Waltham, MA, USA) after acid digestion of dried biomass in a microwave digestion system (Ethos Easy, Milestone Srl, Bergamo, Italy).

The pure C-phycocyanin content in the biomass (% C-PC) was quantified spectrophotometrically at 620 nm after overnight extraction at 4 °C in 100 mM phosphate buffer and calculated using the following equation, as described in [3]:

(3) $$C-PC\;(\%)=\;\frac{{ABS}_{620}\;\times \;10\;\times \;100}{7.3\;\times \;W}$$

where *W* = weight (mg) of moisture- and ash-free dried algal biomass.

Microbiological quality of the packaged samples was assessed by counting the total aerobic mesophilic flora (UNE EN ISO 4833-2 at 30 °C), yeast and mold (ISO 21527), Enterobacteriaceae (ISO 21528-2), total coliforms (NFV 08-050 at 30 °C), *Escherichia coli* (ISO 16649-2), *Staphylococcus* spp. (ISO 6888-2 at 37 °C), and *Clostridium perfringens* (ISO 7937), and by detection of *Salmonella* spp. (ISO 6579) [102].

Total sugars were determined by ion chromatography, while total dietary fiber content was quantified using an enzymatic–gravimetric method.

Fatty acid composition was determined by gas chromatography (GC). The method involves lipid extraction and transesterification to fatty acid methyl esters, which are subsequently separated and quantified using GC with flame ionization detection. Results were expressed as relative percentages of total fatty acids (% *w/w*). Quantification limit was 0.05%. Identification was performed by comparing retention times with certified reference standards.

Amino acids were quantified following Regulation (EU) No. 152/2009. Briefly, proteins were hydrolyzed, and amino acids were identified and quantified chromatographically. Cysteine was determined as cysteic acid after performic acid oxidation. Results are expressed as % of total protein (% *w/w*), with a quantification limit of 0.05%.

Vitamin content was determined according to the following methods: niacin (as nicotinic acid) and vitamin B12 (as cyanocobalamin) were analyzed following USP methods 441 and 171 [118], with quantification limits of 0.08 mg 100 g^−1^ and 0.3 µg 100 g^−1^, respectively. Thiamine (B1, as thiamine hydrochloride) and riboflavin (B2) were quantified by HPLC according to DIN EN 14122:2014-08 and DIN EN 14152:2014-08 methods, with quantification limits of 0.02 mg 100 g^−1^ and 0.05 mg 100 g^−1^, respectively.

### 4.5. Statistical Analysis

Correlation and ANOVA statistical analyses were conducted using PAST 5.2.1 (Paleontological Statistics Software Package for Education and Data Analysis) [119]. Normality and homogeneity of variance were assessed using the Shapiro–Wilk and Levene’s tests, respectively. One-way ANOVA, followed by Tukey’s post hoc test, was applied when assumptions were met, while the Kruskal–Wallis test, followed by Dunn’s post hoc test with Bonferroni correction, was used for non-parametric data. A *t*-test was applied to normally distributed data for pairwise comparisons between shaded and non-shaded culture parameters, metal content between two different phases, and post-harvesting steps of biomass washing and dewatering.

To evaluate the influence of environmental and operational parameters on *L. platensis* productivity and protein, lipid, carbohydrate, and phycocyanin content, a Generalized Linear Model (GLM) was fitted using R software (version 2025.09.1+401, Posit Software, PBC, Boston, MA, USA), employing a Gamma distribution with a log-link function. The GLM model included the following explanatory variables: corrected G_0_, medium recirculation (yes/no), salinity, concentration of KNO_3_, NH_4_H_2_PO_4_, and total carbonate (CO_3_), CO_2_ injection rate, and initial biomass concentration (Cx Start). Because the study was conducted on a single pilot-scale cultivation system monitored sequentially over time, observations are not statistically independent, and phase comparisons may also reflect seasonal variability and long-term operational drift occurring during the experiment. Therefore, the statistical analyses and GLM models were used primarily as exploratory tools to identify associations within the dataset, and their outputs should not be interpreted as evidence of strictly independent causal effects. Moreover, individual semi-continuous cycles were treated as observational units describing the temporal evolution of the same cultivation system, rather than as independent biological replicates.

The dataset included 121 biochemical measurements, 116 C-phycocyanin assessments, 36 microbiological evaluations, and 40 mineral and heavy metal analyses, including replicates, together with a total of 1372 monitoring records of each culture and environmental parameter (Table S1). Statistical significance was set at *p*-value < 0.05 for all analyses, and results were reported as mean ± standard deviation (SD).

### 4.6. Calculations of Energy, Freshwater, and Nutrient Savings

The optimized process of *L. platensis* cultivation in full-seawater with culture medium recycling developed in this study and implemented during phase P6 (hereafter SWR) was compared with the well-established long-term cultivation process of *L. platensis* in freshwater with culture medium recycling ([3,27,35]; hereafter FWR) to estimate the following: (i) energy and freshwater savings along the whole cultivation process (from culture scale-up to fresh biomass harvesting); (ii) fertilizers saved for preparation of the make-up medium (i.e., fresh culture medium); and (iii) the amounts of major inorganic nutrients and ions of environmental concern prevented from being discharged into the environment. Calculations were performed for a case study in the southeast of Gran Canaria for a 10 ha production facility on a yearly basis (360 days operation), assuming 80% productive surface and culture depth of 0.100 m for FWR ([3]; total cultivation volume: 8000 m^3^) and 0.125 m for SWR (this study; total cultivation volume: 10,000 m^3^). In addition to surface-based estimates, results were also normalized per ton (t) of biomass, protein. and phycocyanin produced to account for differences in productivity between cultivation scenarios. The following assumptions were applied:

- An operating culture depth of 0.100 and 0.125 m was adopted in this study for the FWR and SWR scenarios, respectively. A depth of 0.10 m was previously selected to maximize volumetric productivity while reducing freshwater demand for culture medium preparation of *L. platensis* BEA 1257B cultivated in freshwater in the Canary Islands [3]. However, the same authors stated that greater culture depths may enhance areal productivity and total biomass production per unit land area. Since freshwater use for medium preparation was eliminated under full-seawater cultivation, a slightly higher culture depth (0.125 m) was selected in this study to favor areal productivity, and this value was applied in the SWR scenario calculations.
- In both FWR and SWR cultivation processes, culture RWs require an initial fill with the make-up medium. The required water supply is ensured by (i) a reverse-osmosis seawater desalination plant with an electric power consumption (EPC) of 3.91 kWh m^−3^ [106] for FWR; (ii) a submersible pump installed inside a borehole with an EPC of 0.25 kWh m^−3^ for SWR. Fertilizers are supplied according to the specific optimized recipe for each cultivation process (Appendix A.5, Table A2).
- Harvesting of 3000 m^3^ of culture volume is performed weekly for both FWR and SWR: (i) no pumps are used to bring the culture to the vibrating filter, assuming that the harvesting systems are located at a lower level than the cultivation RWs; (ii) the harvest process is performed with vibrating filter units with EPC: 0.50 kWh m^−3^.
- The culture medium after solid–liquid separation by filtration during harvesting (3000 m^3^ week^−1^ for each cultivation process) is pumped back to the culture RWs for volume replenishment (pump EPC: 0.07 kWh m^−3^), except when it is discharged for renewal (see below). Nutrient replenishment after biomass harvesting was assumed to occur with the same amounts of salt reintegrated per kg of harvested dry biomass [3] in both cultivation processes, except for Mg, which is not reintegrated at all in SWR.
- The culture medium renewal rate (RR) has been established as (i) renewal of the entire culture volume every 8 months for FWR (RR: 1.5 year^−1^), taking into account an average value between those reported by [35] (7–12 months) and [27] (6 months). Exhausted medium (effluent) is discharged either to a filtering well or to a sewage treatment plant; (ii) renewal of half of the culture volume every 3 months for SWR (RR: 2 year^−1^), in order to cautiously prevent episodes of milky green coloring of the culture (see Results Section 2.4). Exhausted medium (effluent) is discharged either to a filtering well or to a marine outfall.
- Pump EPC for effluent discharge is 0.07 kWh m^−3^ for both cultivation processes.
- Assumed residual concentrations of N–NO_3_ and P–PO_4_ in the discharged medium are based on literature values for FWR (minimum reported values have been taken into account: N–NO_3_ 181 mg L^−1^; P–PO_4_ 20 mg L^−1^; [27]) and on measurements performed in this study for SWR (maximum measured values have been considered: N–NO_3_ 11 mg L^−1^; P–PO_4_: 5 mg L^−1^). Chloride (Cl^−^) was assumed to be negligibly assimilated by *L. platensis* [52,120].
- The same amount of discharged medium is replaced by make-up medium prepared as outlined above in point 1.
- Daily evaporation has also been considered in freshwater consumption calculations, assuming an evaporation rate of 3.0% day^−1^ for both FWR [3] and SWR (this study). Freshwater used for rinsing the harvested biomass on the vibrating filter (approximately 50 m^3^ per ton of dry biomass produced, see Section 4.3) was not counted as a separate freshwater demand, as this water was entirely returned to the raceway and used to compensate evaporative losses in both cultivation systems (ref. [3] and this study). Consequently, rinsing water is already accounted for within the total freshwater volume required for evaporation compensation.
- Culture mixing with paddlewheels is the same for FWR and SWR. EPC was set at 2.12 kWh m^−2^ year^−1^. This value was estimated by multiplying an average power requirement of 0.245 W m^−2^, calculated as the mean of the values reported by [121] (0.25 W m^−2^) and [122] (0.24 W m^−2^]), by 24 h and 360 days, assuming continuous paddlewheel operation.
- For the normalization of resource savings and nutrient discharge per unit of product, biomass productivity and biochemical composition were assumed based on the cultivation scenarios considered in this study. For FWR, a biomass productivity of 21.9 t ha^−1^ year^−1^ with protein and phycocyanin contents of 62.2 and 7.2 g 100 g^−1^, respectively, was assumed according to [3]. For SWR, a biomass productivity of 14.8 t ha^−1^ year^−1^ with protein and phycocyanin contents of 46.4 and 9.6 g 100 g^−1^, respectively, was assumed based on the results obtained in this study. It should be noted that the FWR benchmark values adopted from [3] represent favorable cultivation conditions obtained during a 1-month medium-recycling period under high nitrate and phosphate concentrations in the culture medium (2 and 0.06 g L^−1^, respectively), whereas the SWR values used here derive from 615 days of operation under reduced nitrate and phosphate supply (0.5 and 0.03 g L^−1^, respectively). Therefore, normalized comparisons should be interpreted in the context of the different cultivation durations and nutrient regimes considered.

## 5. Conclusions

This study demonstrates, for the first time, the long-term outdoor pilot-scale cultivation of *Limnospira platensis* in full seawater with medium recirculation, achieving stable productivity over multiple years and regulatory-compliant effluent discharge without additional treatment under realistic operational conditions.

Under optimized full-seawater recirculation (SWR), biomass productivity (4.1 ± 1.4 gDW m^−2^ day^−1^, 14.8 ± 5.0 t ha^−1^ year^−1^) was 30% lower than under freshwater cultivation of the same strain (FWR, used here as a benchmark), mainly due to the synergistic stress of high irradiance and salinity, and considering the substantially longer recirculation period and lower nutrient supply under SWR conditions. This reduction was more pronounced for protein productivity, while consistently favorable outcomes were observed for phycocyanin. Despite this reduction, biomass quality remained high, with suitable protein quality, high phycocyanin content, essential fatty acids, and a nutritionally relevant mineral composition. Comprehensive safety assessments confirmed compliance with EU food and feed standards, including the absence of heavy metals, pesticides, PAHs, mycotoxins, bacterial pathogens, and seafood-related allergens, supporting its suitability for commercial applications.

From a resource-efficiency perspective, SWR reduced energy demand by 10.5% and freshwater use by 12% on a surface basis, while drastically decreasing fertilizer inputs and the discharge of nutrients and ions of environmental concern, ensuring compliance with effluent regulations. In contrast, FWR offers higher biomass and protein productivity but relies on higher nutrient inputs and generates effluents that would require additional treatment to meet regulatory standards, increasing both operational costs and environmental impact.

Overall, SWR represents a robust and sustainable alternative to conventional freshwater cultivation, particularly suited for arid and semi-arid coastal regions where freshwater availability is scarce or dependent on desalination.

In such contexts, integration with desalination infrastructure offers clear advantages by enabling the use of seawater for cultivation and desalinated water to compensate for evaporation, while the co-discharge of cultivation effluents with desalination brine may help reduce salinity of the final effluent and mitigate environmental impacts. Although further life cycle assessment (LCA) and life cycle costing (LCC) studies are needed to validate the large-scale applicability of this approach, the recent HACCP obtained by the Instituto Tecnológico de Canarias (ITC) further supports its industrial readiness, with ongoing collaborations with local companies for product development and early market introduction.

Future research should focus on improving productivity through optimized cultivation and light management systems, including “aquavoltaic” approaches combining photovoltaic structures for dynamic shading and wind protection with cultivation systems. Strategies to reduce freshwater use for evaporation compensation, including integration with desalination technologies, should also be explored.

## Figures and Tables

**Table 1:** Summary of *L. platensis* culture and environmental parameters across phases P1–P7: number of cultivation cycles per phase (Cycles #), phase duration (Time), and mean ± SD of daily solar radiation (G_0_), temperature (T), pH, salinity (S), CO_2_ supply rate (CO_2_), evaporation rate (ER), and initial and final culture concentrations (Cx Start and Cx End, respectively) at each semicontinuous cycle. Cumulative values of P6.1, P6.2, and P6.3 (P6_Total_) are provided. All phases were carried out in 10,000 L RWs under greenhouse and screen-shaded conditions except for P6NS, where screen shading was not applied.

Phase	P1	P2	P3	P4	P5	P6_Total_	P6NS	P7.CO_2_^+^	P7.CO_2_^−^
Cycles #	13	31	9	38	25	65	38	8	12
Time (days)	50	116	40	217	115	615	314	55	81
G_0_ ^(1)^ (W m^−2^ day^−1^)	*248 ± 46 ^a^* 84 ± 16 ^a^	*310 ± 53 ^b^* 105 ± 18 ^b^	*279 ± 42 ^c^* 95 ± 14 ^a,b,c^	*190 ± 49 ^d^* 64 ± 17 ^d^	*297 ± 58 ^b,c^* 101 ± 20 ^b,e^	*233 ± 70 ^a,e^* 79 ± 24 ^a,c^	*224 ± 73 ^a,e^* 161 ± 52 ^f^	*261 ± 80 ^a,c,f^* 88 ± 27 ^a,c,e^	*278 ± 51 ^a,c,e,f^* 94 ± 11 ^a,c^
T (°C)	22.5 ± 2.0 ^a,b^	24.2 ± 2.3 ^c^	25.0 ± 2.0 ^c^	22.0 ± 2.5 ^a^	23.8 ± 1.8 ^b,c^	22.2 ± 2.7 ^a,d^	22.0 ± 3.1 ^a^	23.8± 2.4 ^b,c,d^	24.7 ± 1.9 ^c^
pH	10.14 ± 0.3 ^a^	10.24 ± 0.3 ^a^	9.73 ± 0.3 ^a,b^	9.48 ± 0.3 ^c^	9.33 ± 0.3 ^c,d^	9.35 ± 0.4 ^d^	9.50 ± 0.4 ^b,e^	9.34 ± 0.3 ^c,d,e^	9.33 ± 0.2 ^c,d^
S (g L^−1^)	10.0 ± 0.6 ^a^	10.3 ± 0.7 ^a^	21.7 ± 1.3 ^a^	38.5 ± 3.9 ^b,(2)^	38.5 ± 1.4 ^b,c^	37.9 ± 1.6 ^c^	36.3 ± 3.0 ^d^	38.1 ± 0.9 ^b,c^	38.1 ± 1.5 ^b,c^
CO_2_ (L min^−1^)	0.19 ± 0.11 ^a^	0.40 ± 0.25 ^b^	0.07 ± 0.04 ^c^	0	0	0	0	0.10 ± 0 ^c^	0
ER (% day^−1^)	3.7 ± 1.5 ^a^	3.7 ± 1.6 ^a^	3.2 ± 1.5 ^a,b^	2.7 ± 1.5 ^b,c^	3.1 ± 1.1 ^a,c^	2.4 ± 1.2 ^b,c^	3.4 ± 1.7 ^a^	2.2 ± 1.1 ^b,c^	2.8 ± 1.4 ^a,b,c^
Cx Start (gDW L^−1^)	0.54 ± 0.1 ^a^	0.94 ± 0.3 ^b,c,d^	0.87 ± 0.2 ^b,c,d^	0.79 ± 0.2 ^b,c^	0.89 ± 0.2 ^b,d^	0.79 ± 0.2 ^b,c^	0.93 ± 0.2 ^d^	0.65 ± 0.1 ^a,b,c^	0.73 ± 0.1 ^a,b,c,d^
Cx End (gDW L^−1^)	0.74 ± 0.1 ^a^	1.2 ± 0.3 ^b^	1.06 ± 0.2 ^b,c,d^	0.95 ± 0.2 ^c,e^	1.06 ± 0.1 ^b,d^	1.05 ± 0.2 ^d^	1.13 ± 0.2 ^b^	0.87 ± 0.1 ^a,e^	0.91 ± 0.1 ^c,e^

^a,b,c,d,e,f^ Letters denote statistically significant differences between experimental phases among the recorded cycles (*p* < 0.05; Dunn’s post hoc test). ^(1)^ Values in italic are full average daily solar radiation measurements. Values in regular correspond to values corrected for radiation attenuation: 66% radiation reduction under greenhouse and screen shading (all phases except for P6NS) and 28% radiation reduction under greenhouse with no screen shading for P6NS. ^(2)^ For P4, mean salinity (±SD) was calculated excluding the 16-day progressive salt acclimation period.

**Table 2:** Culture and environmental parameters recorded for *L. platensis* screen-shaded (P6) and non-shaded (P6NS) cultures within the same temporal framework (7 September 2022 to 19 May 2023, from day 559 to 812). The table includes details on number of semicontinuous cycles per phase (Cycles #), average daily solar radiation (G_0_), temperature (T), pH, salinity (S), evaporation rate (ER), initial culture concentration at each semicontinuous cycle (Cx Start), final culture concentration at each semicontinuous cycle (Cx End).

Phase	Cycles #	G_0_ ^(1)^ (W m^−2^ day^−1^)	T (°C)	pH	S (g L^−1^)	CO_2_ (L min^−1^)	ER (%)	Cx Start (gDW L^−1^)	Cx End (gDW L^−1^)
P6	28	*214 ± 68* 72 ± 23	21.1 ± 2.5	9.23 ± 0.4	38.5 ± 1.2	0	2.1 ± 1.1	0.86 ± 0.21	1.10 ± 0.17
P6NS	33	*214 ± 68* 141 ± 41 *	21.6 ± 3.0	9.53 ± 0.4 *	36.0 ± 3.2 *	0	3.2 ± 1.4 *	0.91 ± 0.19	1.09 ± 0.18

* Denote statistically significant differences between experimental phases among the recorded cycles (*p* < 0.05; *t*-test). ^(1)^ Values in italic are full average daily solar radiation measurements. Values in regular correspond to corrected values for radiation attenuation: 66% radiation reduction under greenhouse and screen shading (for P6) and 28% radiation reduction under greenhouse with no screen shading for P6NS.

**Table 3:** Minimum, maximum, and average volumetric productivity on a dry weight basis (P_vol_) obtained for *L. platensis* BEA 1257B culture phases during semi-continuous cultivation. All phases were carried out under greenhouse and screen-shaded conditions except for P6NS, where screen shading was not applied.

Phase	P1	P2	P3	P4	P5	P6_Total_	P6NS	P7.CO_2_^+^	P7.CO_2_^−^
Min. P_vol_ (gDW L^−1^ day^−1^)	0.040	0.036	0.004	0.012	0.013	0.011	0.010	0.017	0.007
Max. P_vol_ (gDW L^−1^ day^−1^)	0.074	0.134	0.079	0.063	0.121	0.086	0.101	0.047	0.085
Av. P_vol_ (gDW L^−1^ day^−1^)	0.054 ± 0.010 ^a,b^	0.075 ± 0.025 ^a^	0.042 ± 0.023 ^a,b,c^	0.029 ± 0.011 ^c^	0.048 ± 0.026 ^b,d^	0.033 ± 0.014 ^c,d^	0.032 ± 0.021 ^c^	0.031 ± 0.009 ^b,c^	0.032 ± 0.020 ^c,d^

^a,b,c,d^ Letters denote statistically significant differences between experimental phases among the recorded cycles (*p* < 0.05; Dunn’s post hoc test).

**Table 4:** Summary of *L. platensis* BEA 1257B composition, productivity, and environmental parameters during P6 (shaded) and P6NS (non-shaded) phases classified according to pigmentation state. Variables include number of cultivation cycles per phase (Cycles #), C-phycocyanin content (*n* = 4 for P6 and P6NS during pale-green, *n* = 10 for P6 and P6NS during Dark green-blue), productivity (P_vol_), maximum quantum yield (Fv/Fm, λ_ex_ = 630 nm), corrected irradiance (G_0_), and salinity.

	Pale-Green (7 September 2022–30 November 2023)		Dark Green-Blue (1 December 2022–19 May 2023)		Overall (7 September 2022–19 May 2023)	
	P6	P6NS	P6	P6NS	P6	P6NS
Cycles #	10	12	17	21	27	33
PC (% AFDW)	10.7 ± 2.2	6.5 ± 0.5 *	9.0 ± 4.2	6.8 ± 3.9	9.8 ± 3.9	6.7 ± 1.5 *
P_vol_ (gDW L^−1^ day^−1^)	0.037 ± 0.010	0.024 ± 0.015 *	0.026 ± 0.008	0.036 ± 0.020	0.030 ± 0.010	0.031 ± 0.019
Fv/Fm (λ_ex_ = 630 nm)	0.524 ± 0.073	0.428 ± 0.042 *	0.401 ± 0.062	0.438 ± 0.063	0.443 ± 0.088	0.435 ± 0.057
G_0_ (W m^−2^ day^−1^)	72 ± 16	152 ± 35 *	73 ± 26	155 ± 55 *	72 ± 23	154 ± 49 *
Salinity (g L^−1^)	38.9 ± 1.2	38.4 ± 2.4	38.2 ± 1.1	34.8 ± 2.8 *	38.5 ± 1.2	36.0 ± 3.1 *

* Denote statistically significant differences between experimental phases P6 and P6NS among the recorded cycles within each pigmentation state (*p* < 0.05; *t*-test).

**Table 5:** Ash content of *L. platensis* BEA 1257B biomass (expressed in % of dry weight) across the different cultivation phases (P1 to P7).

Phase	P1	P2	P3	P4	P5	P6	P6NS	P7.CO_2_^+^	P7.CO_2_^−^
Ash content (% DW)	13.9 ± 2.6	11.7 ± 2.2	13.5 ± 0.6	13.9 ± 1.4	7.9 ± 5.1	13.5 ± 6.8	12.9 ± 1.9	17.6 ± 9.2	12.8 ± 3.8

Not statistically significant differences between experimental phases among the recorded cycles (*p* > 0.05; Dunn’s post hoc test).

**Table 6:** Mineral composition, trace elements, and heavy metal content in *L. platensis* BEA 1257B biomass samples collected during the different cultivation phases: P1, P3, P4, and P6_Total_. Data are presented as mean ± SD for each phase. “nd” indicates that the element was not detected. “-” indicates that the element was not measured. Minerals and trace elements are expressed as mg 100 g^−1^ DW, whereas heavy metals are expressed as mg kg^−1^ DW due to their different concentration ranges and in accordance with common reporting practices.

Elements		P1	P3	P4	P6_Total_
Minerals (mg 100 g^−1^ DW)	K	1359.5 ± 395.4 ^a^	910.1 ± 124.1 ^b^	1280.2 ± 311.1 ^a^	1645.4 ± 568.7 ^a^
	Na	632.8 ± 153.7 ^a,b^	375.0 ± 97.7 ^a^	1281.3 ± 790.4 ^b,c^	2822.2 ± 2460.4 ^c^
	Mg	243.9 ± 17.9 ^a^	1238.4 ± 92.7 ^b^	742.6 ± 678.5 ^b^	781.6 ± 278.5 ^b^
	Ca	73.6 ± 9.1 ^a^	530.3 ± 223.9 ^b^	248.0 ± 36.5 ^b^	292.8 ± 99.7 ^b^
Trace elements (mg 100 g^−1^ DW)	Fe	58.1 ± 21.2 ^a^	47.3 ± 14.4 ^a^	27.2 ± 7.2 ^b^	32.4 ± 18.1 ^a,b^
	Mn	0.8 ± 0.3 ^a^	4.3 ± 1.2 ^b^	1.3 ± 0.7 ^a,c^	4.7 ± 2.9 ^b,c^
	Cu	0.8 ± 0.1 ^a^	0.4 ± 0.3 ^a,b^	-	0.2 ± 0.2 ^b^
	Zn	0.6 ± 0.2 ^a^	3.3 ± 1.1 ^b^	0.9 ± 0.2 ^a,c^	3.0 ± 1.9 ^b,c^
	B	1.0 ± 0.3 ^a^	4.5 ± 1.2 ^a^	-	4.2 ± 6.4 ^a^
Heavy metals (mg kg^−1^ DW)	Se	nd	3 ± 2 × 10^−3^	-	4 ± 3 × 10^−3^
	Cr	10 ± 2 × 10^−3 a^	28 ± 15 × 10^−3 a^	2 ± 12 × 10^−3 b^	76 ± 67 × 10^−3 a^
	Pb	3 ± 1 × 10^−3 a^	36 ± 8 × 10^−3 b^	19 ± 9 × 10^−3 c^	46 ± 39 × 10^−3 b,c^
	Cd	4 ± 1 × 10^−3^	nd	nd	25 ± 17 × 10^−3^ *
	Mo	5 ± 1 × 10^−3^	nd	-	nd
	As	nd	nd	15 ± 82 × 10^−3^	230 ± 172 × 10^−3^ *
	Hg	nd	nd	nd	nd
	Co	nd	nd	nd	nd

^a,b,c^ Letters denote statistically significant differences between experimental phases among the recorded cycles (*p* < 0.05; Dunn’s post hoc test). * Denote significant differences between experimental phases (*p* < 0.05; *t*-test).

**Table 7:** Microbiological parameters in biomass samples collected during various cultivation phases (P1–P7). Results are expressed as colony-forming units per gram (cfu g^−1^) or as the absence/presence in 25 g. The different lower counting limits are due to the use of different standard methods by the various external analytical laboratories.

Microbiological Parameters	P1	P3	P4	P6_Total_	P6NS	P7.CO_2_^+^	P7.CO_2_^−^
Total aerobic mesophilic flora (cfu g^−1^)	1.2 ± 1.4 × 10^3 a,b^	1.6 ± 0.5 × 10^3 a,b^	6.6 ± 8.1 × 10^3 a,b^	1.1 ± 1.0 × 10^4 a^	2.6 ± 1.5 × 10^2 b^	2.1 × 10^4^	1.4 × 10^4^
Yeasts and molds (cfu g^−1^)	<50	<50	<50	<10 *	<10 *	<10	<100
Enterobacteriaceae (cfu g^−1^)	<50	<50	<50	<10	<10	<100	<100
Total coliforms (cfu g^−1^)	<10	<10	<10	<10	<10	<10	<10
*Escherichia coli* (cfu g^−1^)	<10	<10	<10	<10	<10	<10	<10
*Staphylococcus* spp. (cfu g^−1^)	<50	<50	<50	<10	<10	<100	<10
*Clostridium perfrigens* (cfu g^−1^)	<50	<50	<50	<10	<10	<10	<10
*Salmonella* spp. (Abs 25 g^−1^)	nd	nd	nd	nd	nd	nd	nd

^a,b^ Letters denote statistically significant differences between experimental phases among the recorded cycles (*p* < 0.05; Dunn’s post hoc test); P7.CO_2_^+^ and P7.2CO_2_^−^ were excluded from the analysis due to the availability of a single sample. * For P6_Total_, some samples (*n* = 3) showed values up to 140 cfu g^−1^, whereas in P6NS one sample was 110 cfu g^−1^. “nd“ indicates that the element was not detected.

**Table 8:** Compositional profile of spray-dried *L. platensis* BEA 1257B biomass cultivated under optimized full-seawater conditions (P6_Total_; mean ± SD). General composition is expressed as g 100 g^−1^ of total weight (*n* = 7). Amino acids are expressed as g 100 g^−1^ of the total weight of protein (*n* = 3 except for Asparagine, Cysteine, and Glutamine). Fatty acid content is expressed as % of the total fatty acids (*n* = 6 except for ARA), including high-value fatty acids and those with >1% content. Vitamins are expressed in mg kg^−1^ of biomass (*n* = 2 when SD is included, *n* = 1 in the other cases). Values without standard deviation correspond to measurements obtained from a single sample.

General Composition (g 100 g^−1^)	
Protein	46.42 ± 4.23
Carbohydrates	17.22 ± 7.33
of which sugars	2.3 ± 0.1
of which dietary fiber	7.1 ± 4.0
Lipids	3.41 ± 1.36
of which saturates FA	1.7 ± 0.3
of which monounsaturates FA	0.5 ± 0.1
of which polyunsaturates FA	1.7 ± 0.3
C-phycocyanin	9.64 ± 2.69
Ash	15.63 ± 7.64
Moisture	7.51 ± 1.84
kcal	285
kJ	1193
Amino acid profile (g 100 g^−1^ protein)	
Glutamate	16.62 ± 1.16
Aspartate	9.33 ± 1.53
Leucine	9.09 ± 1.45
Alanine	7.88 ± 1.19
Valine	6.59 ± 0.74
Arginine	6.69 ± 0.92
Isoleucine	5.73 ± 0.79
Glycine	5.11 ± 0.72
Threonine	4.86 ± 1.01
Serine	4.34 ± 1.43
Phenylalanine	4.33 ± 0.76
Lysine	4.31 ± 1.52
Tyrosine	4.27 ± 0.78
Proline	3.52 ± 0.80
Asparagine	2.59
Methionine	1.52 ± 0.44
Histidine	1.44 ± 0.37
Tryptophan	1.25 ± 0.47
Cysteine	0.63
Glutamine	0.04
Fatty acids profile (% of total fatty acids)	
Palmitic acid (16:0)	42.9 ± 0.3
Palmitoleic acid (16:1)	5.4 ± 1.0
Stearic acid (18:0)	1.0 ± 0.1
Oleic acid (18:1)	5.5 ± 2.1
Linoleic acid (LA; 18:2 ω-6)	21.0 ± 1.5
γ-linolenic acid (GLA; 18:3 ω-6)	20.7 ± 1.3
Arachidonic acid (ARA; C20:4 ω-6)	0.12
Σ SFA	44.2 ± 1.2
Σ MUFA	12.5 ± 2.4
Σ PUFA	42.7 ± 2.2
Σ Omega-6 (n-6) fatty acids	42.7 ± 2.2
Σ Omega-9 (n-9) fatty acids	5.5 ± 2.1
Vitamins (mg kg^1^)	
Thiamine (B1)	1.04 ± 0.03
Riboflavin (B2)	3.8 ± 2.4
Niacin (B3)	11.5
Biotin (B8)	2.6 × 10^−3^
Cyanocobalamin (B12)	23.4 ± 23.1 × 10^−3^

**Table 9:** Total and relative savings in energy consumption, freshwater use, and fertilizer requirements resulting from the optimized *L. platensis* full-seawater cultivation process with culture medium recycling developed in this study (SWR) compared with the well-established long-term cultivation process of *L. platensis* in freshwater with culture medium recycling for the make-up medium (FWR; [3,27,35]). Results are expressed on a yearly basis for a 10 ha production facility (80% productive surface) and additionally normalized per ton (t) of biomass, protein, and phycocyanin produced to account for differences in productivity between cultivation scenarios. Cultivation depth was 0.100 m for FWR and 0.125 m for SWR. Daily evaporation rate was 3.0% for both scenarios. Fertilizers were supplied according to the specific optimized recipe for each cultivation process (Table A2). Culture medium renewal rate (RR) was 1.5 year^−1^ for FWR and 2 year^−1^ for SWR. Biomass productivities were assumed to be 21.6 [3] and 14.8 t ha^−1^ year^−1^ (this study), while protein and phycocyanin contents were assumed to be 62.2 and 7.2 g 100 g^−1^ for FWR [3], and 46.4 and 9.6 g 100 g^−1^ for SWR (this study), respectively.

Production Basis	Energy Savings		Freshwater Savings		Fertilizer Savings							
					KNO_3_		NH_4_H_2_PO_4_		CO_3_		NaCl	
	(MWh)	(%)	(m^3^)	(%)	(t)	(%)	(t)	(%)	(t)	(%)	(t)	(%)
Surface-based												
10-ha year^−1^	41.9	10.5	12,000	12.0	14.0 *	58.3 *	0.12 *	16.7 *	23.2 *	67.7 *	60 *	100 *
Product-based												
t biomass	−1.4	−38.1	−171	−30.1	0.1	4.6	−0.001	−1.8	0.1	52.2	0.6	100
t protein	−4.9	−85.1	−680	−74.4	−0.5	−27.8	−0.032	−36.4	0.1	35.9	0.6	100
t phycocyanin	0.9	6.6	266	12.0	1.6	35.5	0.1	31.2	0.5	67.7	1.3	100

Negative values indicate higher resource consumption per unit of product in SWR compared to FWR. * Calculated in the make-up medium.

**Table 10:** Nitrogen as nitrate (N–NO_3_^−^), phosphorus as phosphate (P–PO_4_^3−^), and chloride (Cl^−^) prevented from being discharged (total weight and %) when the optimized *L. platensis* cultivation process in full-seawater with culture medium recycling developed in this study (SWR) is applied, compared with the well-established long-term freshwater cultivation process of *L. platensis* with culture medium recycling (FWR; [3,27,35]). Results are expressed on a yearly basis for a 10 ha production facility (80% productive surface) and additionally normalized per ton (t) of biomass, protein, and phycocyanin produced to account for differences in productivity between cultivation scenarios. Cultivation depth was 0.100 m for FWR and 0.125 m for SWR. Fertilizers were supplied according to the specific optimized recipe for each cultivation process (Table A2). Culture medium renewal rate (RR) was 1.5 year^−1^ for FWR and 2 year^−1^ for SWR. Assumed residual concentrations of N–NO_3_ and P–PO_4_ in the discharged medium were N–NO_3_: 181 mg L^−1^, P–PO_4_: 20 mg L^−1^ for FWR (minimum concentration), and N–NO_3_: 11 mg L^−1^, P–PO_4_: 5 mg L^−1^ for SWR (maximum concentration). Dissolved chloride (Cl^−^) was assumed to be negligibly assimilated by *L. platensis*. Biomass productivities were assumed to be 21.6 [3] and 14.8 t ha^−1^ year^−1^ (this study), while protein and phycocyanin contents were assumed to be 62.2 and 7.2 g 100 g^−1^ for FWR [3], and 46.4 and 9.6 g 100 g^−1^ for SWR (this study), respectively.

Production Basis	Inorganic Nutrients and Ions of Environmental Concern Prevented from Being Discharged					
	N-NO_3_		P-PO_4_		Cl^−^	
	(t year^−1^)	(%)	(t year^−1^)	(%)	(t year^−1^)	(%)
Surface-based						
10-ha year^−1^	1.94	89.6	0.2	86.1	36.4	100
Product-based						
t biomass	0.053	96.5	0.001	79.4	2.08	100
t protein	0.016	79.3	0.002	72.5	0.33	100
t phycocyanin	0.043	89.6	0.005	86.1	0.81	100

Negative values indicate higher resource consumption per unit of product in SWR compared to FWR.

**Table 11:** Chemical compositions of the culture media used for initial maintenance of *L. platensis* BEA 1257B in the growth chamber (OUT medium [3]), and for the subsequent maintenance, indoor scale-up, and outdoor cultivation in greenhouses (OUTs 10% medium with a content of 10% *v/v* of natural seawater).

Chemical	OUT Medium (g L^−1^) [3]	OUTs 10% Medium (g L^−1^) *
NaCl	5	-
NaHCO_3_	8	8
KNO_3_	2	2
NH_4_H_2_PO_4_	0.06	0.06
CO(NH_2_)_2_	0.015	0.015
FeSO_4_·7H_2_O	0.005	0.005
C_6_H_8_O_7_	0.015	0.015
MgSO_4_·7H_2_O	0.16	-
MnCl_2_·4H_2_O	-	0.0015
ZnSO_4_·7H_2_O	-	0.00022
CuSO_4_·5H_2_O	-	0.000025

* Medium recipe used for the first phase (P1) of the present study.

**Table 12:** Summary of the experimental conditions tested for the optimization of *L. platensis* BEA 1257B semi-continuous cultures acclimated to seawater and operated outdoors under a greenhouse in 10,000 L RW. P1 phase: OUTs10% Medium (10% *v/v* seawater); P2 phase: OUTs10% (10% *v/v* seawater) with ¼ nitrogen with respect to P1; P3 phase: OUTs50% (50% *v/v* seawater) with ½ nitrogen, ⅛ bicarbonate, and 1 g L^−1^ carbonate added compared to P1; P4 phase: OUTs100% (full seawater, nutrients like P3); P5 phase: OUTs100% (full seawater), 40% reduced bicarbonates and carbonates with respect to P4; P6_Total_ phase: OUTs 100% (full seawater, subdivided into P6.1, P6.2, and P6.3) with ½ nitrates and phosphates compared to P5; P6NS phase: OUTs 100% (full seawater, nutrient like P6), without shading; P7.CO_2_^+^ and P7.CO_2_^−^ phases (full seawater, nutrients like P6, no medium recirculation, CO_2_ supply, and its absence, respectively).

Experimental Conditions	P1 25 February 2021 –16 April 2021	P2 16 April 2021 –10 August 2021	P3 10 August 2021 –20 September 2021	P4 20 September 2021 –25 April 2022	P5 25 April 2022 –18 August 2022	P6_Total_ P6.1 18 August 2022–19 May 2023 P6.2 2 October 2023 –5 December 2023 P6.3 22 January 2024 –15 October 2024	P6NS 7 September 2022 –28 September 2023	P7.CO_2_^+^ 19 May 2023 –13 July 2023	P7.CO_2_^−^ 13 July 2023 –2 October 2023
Screen shading	√	√	√	√	√	√	X	√	√
Medium Recirculation	√	√	√	√	√	√	√	X	X
CO_2_ supply	√	√	√	X	X	X	X	√	X
Seawater (% *v/v*)	10	10	50	100	100	100	100	100	100
NaHCO_3_ (g L^−1^)	8	8	1	1	0.6	0.6	0.6	0.6	0.6
Na_2_CO_3_ (g L^−1^)	-	-	1	1	0.6	0.6	0.6	0.6	0.6
KNO_3_ (g L^−1^)	2	0.5	1	1	1	0.5	0.5	0.5	0.5
NH_4_H_2_PO_4_ (g L^−1^)	0.06	0.06	0.06	0.06	0.06	0.03	0.03	0.03	0.03

√ indicates that the experimental condition was applied; X indicates that the experimental condition was not applied.

## Data Availability

Data is contained within the article or Supplementary Materials.

## Supplementary Materials

The following supporting information can be downloaded at: https://www.mdpi.com/article/10.3390/md24040141/s1, Table S1: Data Availability Section.

## Appendix A

### Appendix A.3

**Table A1:** Dissolved nutrient concentrations (NO_3_^−^ and PO_4_^3−^) before harvesting and after medium replenishment across the different cultivation phases. Measured values were determined using semi-quantitative test strips (Quantofix^®^ test strips).

	Nutrient Concentration (mg L^−1^)							
	Before Harvesting				After Replenishment			
Phase	NO_3_ Target	NO_3_ Measured	PO_4_ Target	PO_4_ Measured	NO_3_ Target	NO_3_ Measured	PO_4_ Target	PO_4_ Measured
P1	≤50	900 ± 329	≤10	11 ± 7	1200	1200 ± 0	50	50 ± 0
P2	≤50	27 ± 60	≤10	4 ± 3	300	326 ± 109	50	50 ± 0
P3	≤50	240 ± 82	≤10	19 ± 8	600	600 ± 0	50	50 ± 0
P4	≤50	244 ± 79	≤10	22 ± 6	600	600 ± 0	50	50 ± 0
P5	≤50	99 ± 46	≤10	11 ± 6	600	600 ± 0	50	50 ± 0
P6_Total_	≤50	28 ± 18	≤10	3 ± 4	300	300 ± 0	25	25 ± 0
P7.CO_2_^+^	≤50	46 ± 9	≤10	9 ± 3	300	300 ± 0	25	25 ± 0
P7.CO_2_^−^	≤50	42 ± 28	≤10	6 ± 4	300	300 ± 0	25	25 ± 0
P6NS	≤50	26 ± 17	≤10	7 ± 4	300	300 ± 0	25	25 ± 0

### Appendix A.5

**Table A2:** Chemical compositions of the culture media used for cultivation of *L. platensis* BEA 1257B in the FWR scenario (OUT medium, freshwater-based medium [3]) and in the SWR scenario (OUTs100%, full-seawater-based medium, P6_Total_) and estimated nutrient replenishment for both scenarios.

Chemical	FWR OUT Medium (g L^−1^) [3]	SWR OUTs 100% Medium (g L^−1^) [this study]	Estimated Nutrient Replenishment (g kg^−1^ of Algal DW)
NaCl	5	-	-
NaHCO_3_	8	0.6	-
Na_2_CO_3_	-	0.6	-
KNO_3_	2	0.5	1000 [3]
NH_4_H_2_PO_4_	0.06	0.03	50 [3]
CO(NH_2_)_2_	0.015	0.015	*
FeSO_4_·7H_2_O	0.005	0.005	*
C_6_H_8_O_7_	0.015	0.015	*
MgSO_4_·7H_2_O	0.16	-	30 **
MnCl_2_·4H_2_O	0.0015	0.0015	*
ZnSO_4_·7H_2_O	0.00022	0.00022	*
CuSO_4_·5H_2_O	0.000025	0.000025	*

* reintegrated proportionally to nitrate supplementation, ** only replenished in FWR.
